# A novel microRNA signature predicts survival in stomach adenocarcinoma

**DOI:** 10.18632/oncotarget.15961

**Published:** 2017-03-07

**Authors:** Bowen Ding, Xujie Gao, Hui Li, Liren Liu, Xishan Hao

**Affiliations:** ^1^ Department of Gastrointestinal Cancer Biology, National Clinical Research Center of Cancer, Tianjin Medical University Cancer Institute and Hospital, Tianjin 300060, China

**Keywords:** miRNA signature, prognostic biomarkers, overall survival, stomach adenocarcinoma

## Abstract

Recent microRNA (miRNA) expression profiling studies suggest the clinical use of miRNAs as potential prognostic biomarkers in various malignancies. In this study, aiming to identify microRNAs with prognostic value for overall survival (OS) in stomach adenocarcinoma (STAD) patients, we analyzed the miRNA expression profiles and the associated clinical characteristics in 380 STAD samples from The Cancer Genome Atlas (TCGA) dataset. An eight-miRNA signature for predicting OS in STAD patients was identified and self-validated by survival analysis and semi-supervised principal components method. We developed a linear prognostic model composed of these miRNAs to divide patients into high- and low-risk groups according to the calculated prognostic scores. Kaplan-Meier analysis demonstrated that patients in the high-risk group had worse OS compared with patients in the low-risk group. Notably, this miRNA prognostic model showed prognostic significance to the STAD patients in early stages and the chemo-resistant patients, who would potentially benefit from additional medical interventions. Finally, this eight-miRNA signature is an independent prognostic biomarker and demonstrates a good predictive performance for 5-year survival. Thus, this signature may serve as a novel biomarker for predicting survival as well as chemotherapy response in STAD patients.

## INTRODUCTION

Gastric cancer (GC) represents a major health problem worldwide, ranking the fifth most common malignancy and the third leading cause of global cancer mortality [1]. Early diagnosis is critical to the prognosis of stomach adenocarcinoma (STAD) patients, which constitute the majority (> 90%) of GC patients. Unfortunately, most STAD patients are diagnosed at advanced stage disease which is manifested by extensive tumor invasion and/or distant metastasis, and results in a poor 5-year survival rate with the median survival of less than 1 year [2–4]. Over the past few decades, despite significant progress in diagnostics and therapeutics, the overall outcomes of GC patients has only modestly improved [5]. Thus, it is essential to discover specific prognostic factors that may help guide the clinical therapeutic implications and improve the overall survival (OS) for these patients.

In recent years, various miRNAs and their targets have been found to be dysregulated in cancer. Due to the stability and expression specificity of miRNAs in different tissues, there is increasing evidence to suggest that they can serve as novel prognostic biomarkers with potential clinical significance for various cancer types. Thus far, dysregulation of miRNAs in GC has been reported to be associated with histology, Epstein-Barr virus infection, chemotherapy, and metastasis [6–11]. A number of miRNAs were further proposed to be potential prognostic biomarkers for outcomes of GC patients, such as miR-20b, miR-150, miR-214, miR-375, Let-7g, miR-125-5p, miR-146a, miR-218, miR-433, miR-451 and miR-200b/c [6, 12–14] [15–19]. Moreover, a seven-miRNA signature (miR-10b, miR-21, miR-223, miR-338, let-7a, miR-30a-5p, miR-126) has recently been identified as an independent predictor for relapse-free survival of GC patients [16]. However, the miRNAs characterized from these studies showed a wide range of diversity and inconsistency, which may be primarily due to the fact that these results were mostly derived from the analysis based on microarray data. As we know, besides the limitations in the dynamic range, sensitivity, and specificity of the microarray data themselves, different technological detection platforms, small sample size, various methods employed for data processing and analysis may result in significant variations.

With the advent of next-generation sequencing and bioinformatics as well as the launches of large-scale cancer genome projects, such as The Cancer Genome Atlas (TCGA), comprehensive and multi-dimensional maps of the key genomic and epigenomic changes in cancer can be readily achieved and accessed. Bioinformatic analysis of TCGA datasets has been shown to be an outstanding tool in identifying genetic and epigenetic changes related to clinical outcomes, thus opening a new avenue for the discovery of novel prognostic markers for various malignancies [20, 21]. In this study, aiming to identify a panel of specific miRNAs associated with OS in STAD patients, we applied the bioinformatics analysis based on the genome-wide miRNA-Seq profiles derived from TCGA dataset, which represents the largest STAD cohort available up to date. To minimize the variations among different individuals, we first characterized the differentially expressed miRNAs by comparing miRNA profiles in paired STAD and normal tissues. We then developed a linear prognostic model composed of eight miRNAs to divide patients into high- and low-risk groups according to their calculated prognostic scores. The following Kaplan-Meier analysis demonstrated that the eight-miRNA signature correlated with a good predictive performance for 5-year survival and chemotherapy response in STAD patients, suggesting that this signature may serve as a novel biomarker to complement the traditional histopathological prognostic factors and help guide individual therapy for these patients.

## RESULTS

### Differentially expressed miRNAs in STAD versus paired adjacent normal tissue

After filtering out unqualified cases, miRNA expression and clinical data of 380 STAD patients were retained for survival analysis. It contained 252 male and 128 female, among all the participants a total of 41 patients with adjacent non- tumor tissues. The patients were randomly divided into the training set (*n* = 190) and testing set (*n* = 190). No significant difference in clinical covariates was observed between the two sets (Table 1).

**Table 1:** Clinicopathological characteristics of the study cohort

Variable	Total (*n* = 380)	Training Set (*n* = 190	Testing Set (*n* = 190)	*P*
Age(year) < 60 ≥ 60	120 (31.6%) 260 (68.4%)	67 (35.3%) 123 (64.7%)	53 (27.9%) 137 (72.1%)	0.122 *
Sex Male Female	252 (66.3%) 128 (33.7%)	124 (65.3%) 66 (34.7%)	128 (67.4%) 62 (32.6%)	0.664
Vital status Alive Dead	230 (60.5%) 150 (39.5%)	115 (60.5%) 75 (39.5%)	115 (60.5%) 75 (39.5%)	1.000
Stage I II III IV	47 (12.4%) 132 (34.7%) 182 (47.9%) 19 (5.0%)	24 (12.6%) 67 (35.3%) 92 (48.4%) 7 (3.7%)	23 (12.1%) 65 (34.2%) 90 (4.74%) 12 (6.3%)	0.708
T stage T1 T2 T3 T4	20 (5.3%) 71 (18.7%) 126 (33.1%) 163 (42.9%)	10 (5.3%) 36 (18.9%) 61 (32.1%) 83 (43.7%)	10 (5.3%) 35 (18.4%) 65 (34.2%) 80 (42.1%)	0.987
N stage N0 N1 N2 N3	118 (31.0%) 106 (27.9%) 74 (19.5%) 82 (21.6%)	61 (32.1%) 51 (26.8%) 40 (21.1%) 38 (20.0%)	57 (30.0%) 55 (28.9%) 34 (17.9%) 44 (23.2%)	0.750
M stage M0 M1	357 (93.9%) 23 (6.1%)	181 (95.3%) 9 (4.7%)	176 (92.6%) 14 (7.4%)	0.282
Adjuvant treatment None Chemotherapy Radiotherapy Chemoradiotherapy	166 (58.9%) 153 (40.3%) 3(0.8%) 58 (15.3%)	83 (43.7%) 79 (41.5%) 2(1.1%) 26 (13.7%)	83 (43.7%) 74 (38.9%) 1 (0.6%) 32 (16.8%)	0.993

Analysis of miRNA expression profiles in 41 pairs of STAD and normal tissues identified 138 differentially expressed miRNAs (logFC > 1 or logFC < −1, *P* < 0.05 after FDR adjustment). Among these, 77 miRNAs (55.8%) were up-regulated, including miR-1269 and miR-196a-1, which exhibited > 5-fold increased expression. Conversely, 61 miRNAs (44.2%) were down-regulated, including miR-490 and miR-1-2, which showed 4.8-fold and 3.5-fold reduced levels in STAD, respectively (Supplementary Table 1).

### Establishment of the miRNA prognostic model

Using univariate Cox regression, we characterized the common miRNAs that were associated with OS within each of the following independent subclass: differentiation, pathologic N stage, pathologic T stage, and pathologic M stage. Within each subset of clinical characteristics, the patient subclasses represented non-overlapping sets, respectively. MiRNAs were selected if they were associated significance with OS in at least two independent subclasses. The respective HRs for the association of miRNA with OS in each subclass were shown in Table 2. Seventeen miRNAs were identified in this analysis.

**Table 2:** MiRNAs associated with prognosis in different clinical subclasses

miRNA	T1-2 HR (95% CI)	T3-4 HR (95%CI)	N0 HR (95% CI)	N1-3 HR (95% CI)	M0 HR ( 95% CI)	M1 HR (95% CI)	Grade1-2 HR (95% CI)	Grade3 HR (95% CI)
miR-100	1.31 (1.03–1.66)	-	-	1.20 (1.03–1.40)	1.21 (1.05–1.40)	-	-	-
miR-125a	1.58 (1.10–2.28)	1.29 (1.00–1.67)	-	1.40 (1.10–1.78)	1.32 (1.06–1.63)	-	1.72 (1.23–2.39)	-
miR-125b-1	1.30 (1.02–1.65)	-	-	-	1.17 (1.01–1.34)	-	-	-
**miR-145**	-	-	-	1.16 (1.01–1.34)	1.16 (1.02–1.31)	-	1.25 (1.01–1.56)	-
**miR-1537**	-	-	-	0.79 (0.68–0.93)	0.84 (0.72–0.98)	-	-	-
**miR-184**	-	-	-	-	-	1.23 (1.02–1.46)	1.18 (1.04–1.34)	-
**miR-20b**	-	1.14 (1.01–1.30)	1.30 (1.07–1.57)	-	-	-	1.19 (1.01–1.41)	-
miR-28	-	1.45 (1.06–1.98)	-	-	-	-	1.68 (1.04–2.71)	-
miR-30a	-	1.22 (1.03–1.51)	-	-	1.19 (1.02–1.40)	-	-	-
miR-328	-	1.25 (1.03–1.51)	-	1.25 (1.05–1.49)	1.19 (1.02–1.39)	-	1.33 (1.05–1.69)	-
miR-365-1	-	-	1.72 (1.08–2.74)		-	-	1.54 (1.04–2.27)	-
miR-383	-	-	1.33 (1.07–1.66)		-	-	1.24 (1.01–1.52)	-
**miR-549**	0.79 (0.63–0.98)	-	0.73 (0.55–0.98)		0.87 (0.76–0.99)	-	-	-
**miR-802**	-	-	-		0.88 (0.77–0.99)	-	-	0.85 (0.73–0.99)
**miR-9-1**	-	1.14 (1.02–1.26)	1.22 (1.00–1.48)	-	1.10 (1.00–1.20)	--	-	1.15 (1.02–1.31)
**miR-9-2**	-	1.14 (1.03–1.27)	1.21 (1.00–1.47)	-	1.10 (1.00–1.20)	--	-	1.16 (1.02–1.31)
miR-99a	1.23 (1.00–1.50	-	-		1.13 (1.01–1.26)	-	-	-

**Figure 1:**
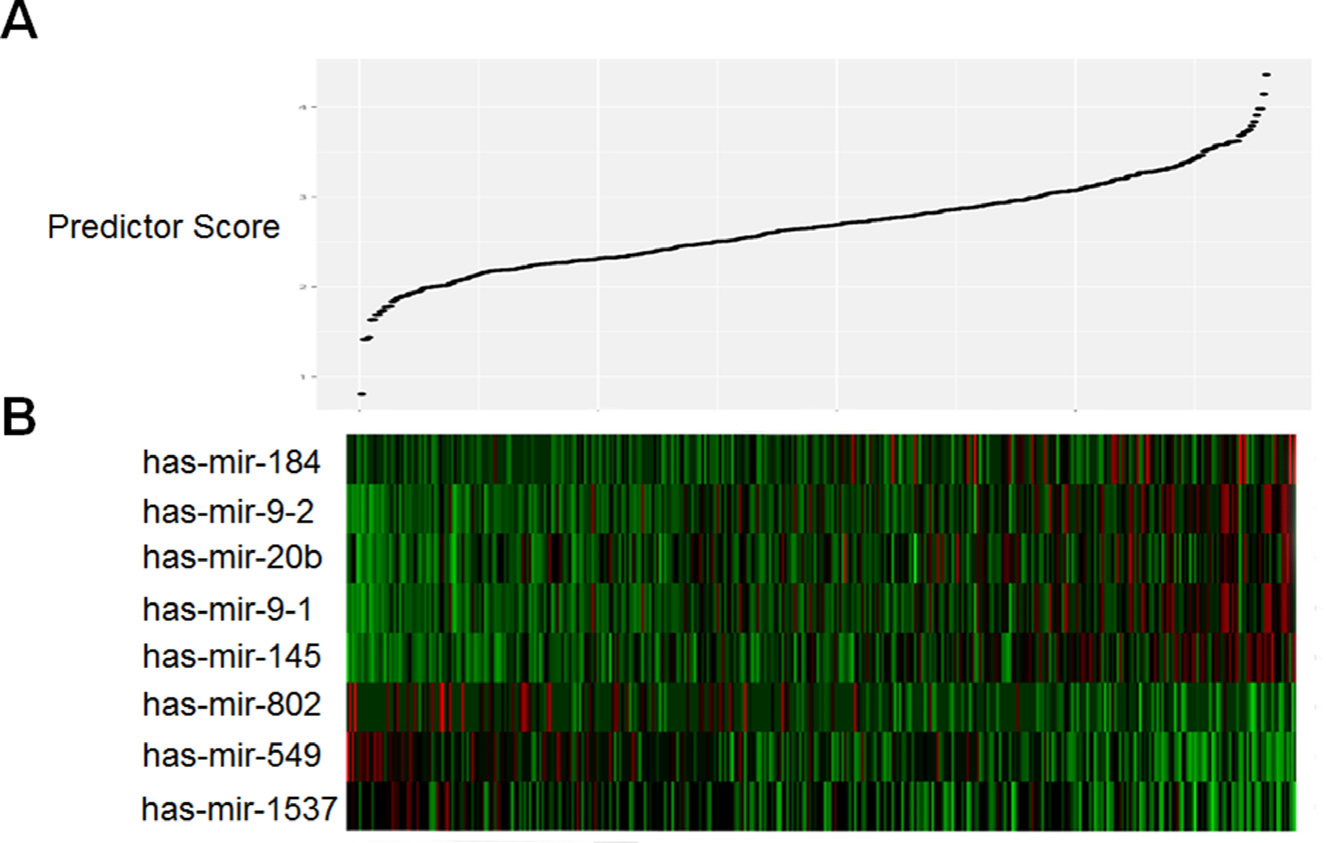
Heatmap and predictor-score of eight-MicroRNA signature of STAD cohort (**A**) MicroRNA predictor-score distribution. (**B**) Heatmap of eight miRNAs expression profiles of STAD patients.

Eight of 17 miRNAs were selected using the supervised principal component method in the training set. Among these eight miRNAs, five were associated with high risk (mir-145, mir-184, miR-20b, miR-9-1 and miR-9-2, HR > 1) and three were shown to be protective (miR-1537, miR-549 and miR-802, HR < 1). We then developed a miRNA prognostic model for predicting 5-year survival in the training set, by which the samples were classified into high-risk or low-risk groups using the optimum cutoff point of miRNA scores according to ROC curve. Figure 1 showed the distribution of patient prognostic scores and miRNA expression of all 380 STAD patients, ranked by the prognostic score values for the eight-miRNA signature. Patients with high prognostic scores tended to express high-risk miRNAs, whereas patients with low prognostic scores tended to express protective miRNA s (Figure 1A and 1B).

### Validation of the eight-miRNA signature in STAD patients

**Figure 2:**
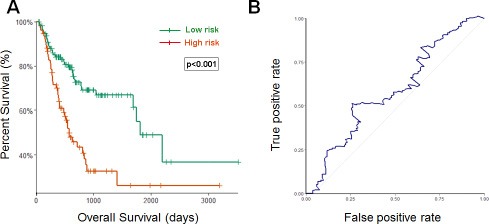
Kaplan–Meier and ROC curves for the eight-miRNA signature in STAD testing set (**A**) The Kaplan–Meier curves for testing set (*n* = 190) divided by the optimum cutoff point. Patients with high scores had the poor outcome in terms of OS (Median OS: 1811days vs. 570 days, *p* < 0.001). (**B**) The ROC curve for predicting 60-month survival for testing set.

**Figure 3:**
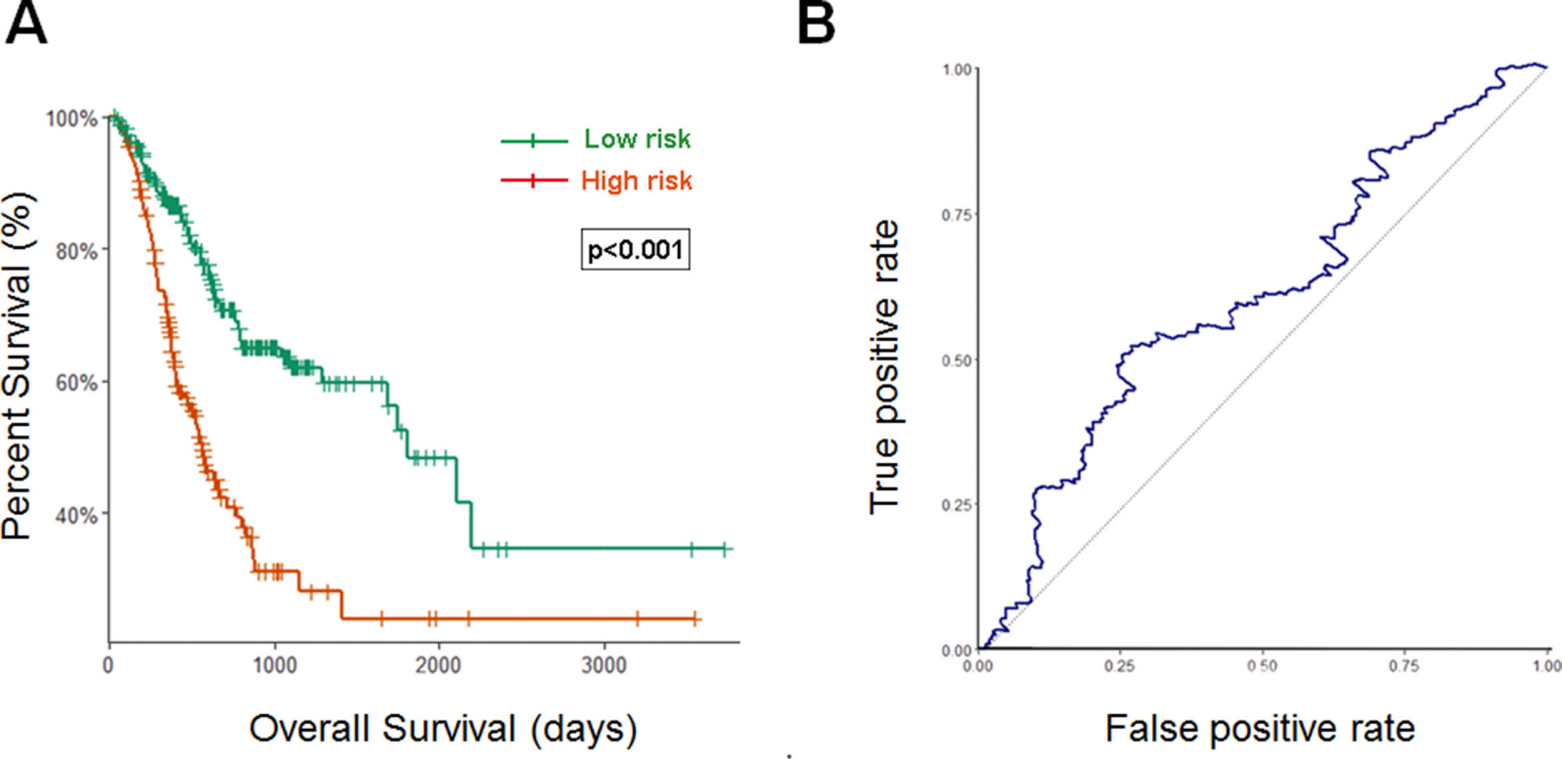
Kaplan–Meier and ROC curves for the eight-miRNA signature in STAD cohort (**A**) The Kaplan–Meier curves for entire STAD cohort divided by the optimum cutoff point. Patients with high scores had the poor outcome in terms of OS (Median OS: 1811 days vs. 562 days, *p* < 0.001). (**B**) The ROC curve for predicting 60-month survival for STAD cohort.

Using the optimum cutoff value obtained from the training set, patients were assigned into high-risk and low-risk groups. The ability of prognostic prediction of the eight-miRNA signature was examined in the testing set and the entire STAD cohort, respectively. Kaplan-Meier analysis revealed that patients in the high-risk group had poor OS, compared with patients in the low-risk group in both testing set (*p* < 0.001, using the log-rank test, Figure 2A) and the entire STAD cohort (*p* < 0.001, using the log-rank test, Figure 3A). Time-dependent ROC curves were also used to assess the prognostic power of the eight-miRNA signature. The AUC of the eight-miRNA signature prognostic model for the testing set and the entire STAD cohort was 0.586 (Figure 2B) 0.607 (Figure 3B), respectively.

**Figure 4:**
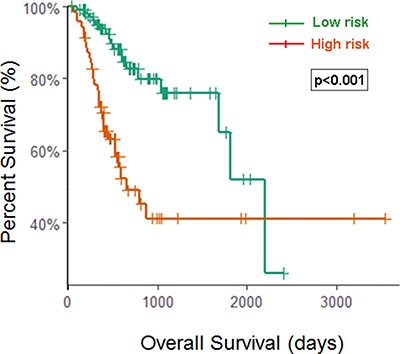
Kaplan–Meier curves for the eight-miRNA signature in early stage patients Patients with high scores had poor outcome in terms of OS (Median OS: 2197 days vs. 652 day, *p* < 0.001).

Since patients with early stage disease may benefit significantly from a prognostic biomarker signature, we evaluated the prognostic power of the eight-miRNA signature in stage I and II patients (*n* = 179). Kaplan-Meier analysis revealed that patients in the high-risk group associated with worse OS (*p* < 0.001, using the log-rank test). Thus, this eight-miRNA signature could distinctly predict the survival for STAD patients in an early stage, suggesting that it could have potential clinical value for help in guiding additional medical interventions to this patient subgroup. (Figure 4)

It is often challenging to distinguish chemoresistant STAD patients from those that have good responses to chemotherapy. In order to evaluate the prognostic value of the eight-miRNA signature to chemotherapy response in STAD patients, we applied the eight-miRNA signature to the 112 patients, whose post-operative chemotherapy responses were recorded in the TCGA STAD cohort. Using our eight-miRNA prognostic model, the 112 patients were divided into low-risk and high-risk subgroups according to their responses to chemotherapy. As shown in Table 3, among 68 low-risk patients, most patients had either a complete or partial response after chemotherapy, while only 7 patients in this subgroup showed disease progression. In contrast, among 44 high-risk patients, approximately one-third of patients showed progression after chemotherapy. Thus, this eight-miRNAs signature showed a good prognostic power for predicting chemotherapy response in STAD patients (*P* value < 0.05).

**Table 3:** Association of eight-miRNA signature with chemotherapy response

miRNA signature (#)	CR&PR	SD	Progress	*p*-value
Low-risk (68)	55	6	7	0.017
High-risk (44)	27	3	14	

CR: complete response, PR: partial response, SD: stable disease.

We also examined the association of eight-miRNA signature with clinical characteristics in STAD patients. No significant differences were observed when patients were stratified by gender and age (Supplementary Table 2).

### The eight-miRNA signature is an independent prognostic factor

We further conducted a multivariate analysis to evaluate the independent prognostic value of the eight-miRNA signature. Age, gender, Grade, T stage, N stage, M stage and the miRNA signature were used as covariates. The Cox multivariate regression analysis revealed that the miRNA signature is an independent prognostic factor associated with OS (Table 4; HR = 2.003, *p* < 0.001).

**Table 4:** Multivariate analysis of overall survival of patients

Characteristic	HR (95% CI)	*P* value
Gender (male vs. female)	0.781 (0.551–1.107)	0.165
Age (< 60 vs. ≥ 60 years)	1.719 (1.186–2.490)	**0.004**
Grade (Grade 1–2 vs Grade 3)	1.315 (0.928–1.863)	0.123
T stage (T1–2 vs T3–4)	1.417 (0.896–2.240)	0.136
N stage (N0 vs N1–3)	1.786 (1.184–2.695)	**0.006**
M stage(M0 vs M1)	2.346 (1.328–4.146)	**0.003**
miRNA signature	2.003 (1.458–2.752)	**< 0.001**

### *In silico* analysis of target genes and pathways

The list of predicted target genes of these eight miRNAs was downloaded from miRecords. A total of 3510 target genes which predicted by more than 5 data sets were identified to be potentially regulated by the eight miRNAs. We then performed a functional enrichment analysis to elucidate the biological function of these target genes by Kyoto Encyclopedia of Genes and Genomes (KEGG) categories and Gene Ontology (GO) categories. As shown in Supplementary Table 3, the analysis revealed enrichment of 45 KEGG categories and 714 GO categories (*P*-values < 0.05 after FDR adjustment), demonstrating the predicted miRNA targets are involved in many important pathways associated with cancer development, including adherent junction, Wnt, TGF-beta, VEGFR and MAPK signaling pathways (Table 5). These results highlighted critical roles of these eight miRNAs in STAD onset and progression, and the underlying mechanisms warrant further investigation.

**Table 5:** Results of pathway analysis of the target genes

Pathways	Target genes
Wnt signaling pathway	CAMK2D, CCND2, CTNNBIP1, LRP6, PPP3CA, PRKX, ROCK1, SENP2, SMAD3, VANGL1
MAKP signaling pathway	CRK, CRKL, ELK4, FLNB, MAP4K2, PAK1, PLA2G4A, PPP3CA, PRKX, RAPGEF2, RASA1, TAOK1, TGFBR2
Adherens junction	ACTB, ACTG1, IGF1R, SMAD3, TGFBR2, TJP1, YES1
TGF-beta signaling pathway	ACVR1B, ACVR2A, ROCK1, SMAD3, SMAD5, TGFBR2, ZFYVE9
VEGF receptor signaling pathway	ARNT, NEDD4, LECT1, BMPR2, HIF1A, FLT1, VEGFA, HHEX, GRB10, FOXC1

## DISCUSSION

MiRNAs are 22–26 nucleotides small RNAs that play vital roles in modulating gene expression at the post-transcriptional level by binding to the 3′ or 5′ untranslated region of targeted mRNAs. Here we found 138 miRNAs were differentially expressed between STAD and adjacent normal tissues, among which 17 miRNAs levels were associated with at least two of following histopathological factors: T stage, lymph node metastasis, distant metastasis and tumor grade. Using survival analysis and semi-supervised principal components method, an eight-miRNA signature (miR-145, miR-184, miR-20b, miR-9-1, miR-9-2, miR-1537, miR-549 and miR-802) for predicting OS of STAD patients was identified and self-validated in a large cohort. Importantly, this eight-miRNA signature was further confirmed to be an independent prognostic factor. Furthermore, with respect to the association between their expression levels and patient survival, the eight miRNAs in the signature were divided into two groups: five risky miRNAs that were negatively associated with the survival and three protective miRNAs which were positively associated with the survival.

Among the five risky miRNAs (mir-145, mir-184, miR-20b, miR-9-1 and miR-9-2), overexpression of miR-145 has been reported to be a key factor for the prediction of poor overall survival in bladder cancer [22]. However, a recent study based on qRT-PCR showed that downregulation of miR-145 correlates with a poor survival in a clinical cohort composed of 145 GC patients from the north of China, which is inconsistent with our result that mir-145 was identified as one of the five risky miRNAs leading to poor 5-year survial. The discrepancy among these studies could be because there are distinct expression patterns of miR-145 in either different cancer types or the same cancer type with subtle variations, such as race. Upregulation of miR-184 enhanced the malignant phenotype of glioma cancer cells by reducing FIH-1 protein expression and facilitated the proliferation and invasion in pancreatic ductal adenocarcinoma [23, 24]. Overexpression of miR-20b was reported to be associated with poor outcomes in GC patients [6, 25]. Also, high level of miR-20b facilitated brain metastasis of breast cancer and promoted hepatocellular carcinoma invasion and progression [26, 27]. The role of miR-9 has been investigated in many types of malignancy, but the results were inconsistent and inconclusive. Recently, a systematic meta-analysis, representing a quantified synthesis of all published studies of miR-9, found that high expression of miR-9 was significantly associated with poor survival in patients with malignancies, including colorectal cancer, non-small lung cancer, breast cancer, hepatocellular carcinoma, laryngeal and esophageal squamous cell carcinomas, glioma, ovarian, osteosarcoma, adrenocortical cancer, bladder cancer, and leukemia [28].

Among the protective miRNAs (miR-1537, miR-549 and miR-802), deletion of mir-1537 has been reported in aggressive neuroblastoma [29]. During neoadjuvant therapy in the patients with advanced adenocarcinomas of the gastroesophageal junction, there was a significant increase of serum miR-549, suggesting a suppressive role it played in tumor progression [30]. Downregulation of miR-802 was significantly associated with overall survival and cancer-specific survival in rectal cancer patients [31]. Taken together, these biological and clinical studies of the miRNAs have provided some insights into their potential prognostic value, although future work is needed to validate their roles in clinical applications.

It is of note that this eight-miRNA prognostic model could predict the high-risk patients in early stages (stage I–II), which accounts for approximately 10% of all STAD recurrences [32]. These patients may benefit significantly from a prognostic biomarker to guide their further medical interventions, such as the shorter interval between the exams for monitoring the recurrence or alternative treatment approaches. Since chemotherapy resistance is one of the major factors leading to a poor OS for STAD patients, we applied our miRNA signature for the prediction of chemotherapy response. Indeed, the chemoresponsive or chemoresistant patients were well distinguished using this eight-miRNA signature, which made it possible in clinical practice to stratify patients into two groups: one that could benefit from standard therapy and another that should be placed on alternative therapeutic protocols or novel clinical trials.

Bioinformatic analysis of potential and validated targets of miRNA and the subsequent analysis of KEGG pathways clustered by target genes are promising strategies for gaining insights into plausible biomarkers and key events involved in cancer development and progression. Enriched KEGG pathway analysis of the eight-miRNA model indicated that both predicted and validated target genes of this miRNA signature were clustered in cancer-associated KEGG pathways. Furthermore, our *in silico* analysis proved that the eight-miRNA signature is biologically meaningful. Specifically, Ago2, the co-target of miRNA-145, miR-184 and miR-802, played an important role in GC differentiation, lymph node invasion and clinical stage [33]. Myc, a multiplayer in carcinogenesis, progression and metabolism, was also the predicted target of miR-145 and miR-184 [34]. Moreover, many target genes related to VEGF receptor signaling pathway have been identified, such as VEGFR-3. Indeed, high expression level of VEGFR-3 has been found to be associated poor survival of gastric adenocarcinoma [35]. Certainly, further molecular biological experiments need to be performed to validate these predictions.

In summary, using integrated bioinformatic analysis of the largest available cohort of STAD patients and the corresponding genome-wide miRNA sequencing results, we identified a specific eight-miRNA signature that could serve as an independent prognostic factor for the prediction of OS in STAD patients. However, before this finding can be applied to clinical practice, further validation in independent large cohorts is required in the future.

## MATERIALS AND METHODS

### TCGA dataset

Level 3 of 1049 miRNAs expression profiles in stomach adenocarcinoma (STAD) patients and their clinical information dataset were downloaded from The Cancer Genome Atlas (TCGA) data portal (March 2016). To exclude unrelated causes of death, only patients with follow-up longer than 1 month were included in the subsequent analysis.

### Identification of dysregulated miRNAs in STAD

The raw counts of miRNA expression data of 41STAD with their paired normal tissue were obtained from the TCGA dataset (Illumina HiSeq Systems). MiRNA-expression data was normalized by the R/Bioconductor package edgeRv [36]. The expression differences were characterized by logFC (log2 fold change). MiRNAs with logFC < −1 or logFC >1 (FDR-adjusted *p* < 0.05) were considered as differentially expressed miRNAs and were selected for further analysis.

### Identification of miRNAs with prognostic value in STAD

The semi-supervised method which combines both the gene expression data and the clinical data was used to identify candidate miRNAs with prognostic value [37, 38]. Univariate Cox regression analyses were conducted to identify common miRNAs related to OS within each of the subgroups stratified by the TMN stage. Within each group of clinical characteristics, the patient subclasses represented non-overlapping sets. Common miRNAs associated with OS in at least two independent subgroups were selected for the subsequent studies, using an HR>1 or HR<1 with *p* < 0.0.5 as the cutoff.

### Definition of prognostic risk model and ROC curve analysis

An importance score was calculated by the supervised principal components method and was assigned to each miRNA [37]. Ten-fold cross-validation was used to estimate the optimal feature threshold in supervised principal components and to select significant miRNAs. The TCGA dataset was randomly divided into the training set and the testing set. The linear miRNA signature prognostic model was developed based on the supervised principal component method. The miRNAs expression level was as the log2 reads per million of total aligned miRNA reads. The prognostic score was calculated as follows: Prognostic-score = (0.1482 × miR-145) + (−0.0987 × miR-1537) + (0.1126 × miR184) + (0.0964 × miR20b) + (−0.1662 × miR-549) + (−0.1374 × miR-802) + (0.0915 × miR-9-1) + (−0.0148 × miR-9-2). Then, the prognostic scores were computed for the 380 patients using our miRNA prognostic model. The best cutoff values of the prognostic score were decided in the ROC curve analysis for predicting 5-year survival of the training set. The OS curves were evaluated using the Kaplan–Meier and log-rank method. Time-dependent ROC curves were used to evaluate the predicted power of the miRNAs signature model. All analyses were performed using the R/Bioconductor (version 3.3.1).

### Bioinformatic analysis of miRNA-target genes and pathways

The list of predicted target genes of the candidate miRNAs was obtained from miRecords v4.0 (www.mirecords.biolead.org) database, which offers a comprehensive data of possible miRNA-targets of 11 different data sets. The pathway enrichment analysis was conducted with the GeneTrail gene set enrichment tool. The results were considered significant when the *p*-value was less than 0.05 after FDR corrected [39, 40].

## SUPPLEMENTARY MATERIALS FIGURES AND TABLES






