# Supplementary material for: A novel microRNA signature predicts survival in stomach adenocarcinoma
Source: Oncotarget. 2017 Mar 7;8(17):28144–53. doi: 10.18632/oncotarget.15961 (PMC5438638; doi:10.18632/oncotarget.15961)
Supplement: Supplementary file 1 [file oncotarget-08-28144-s001.pdf]

A novel microRNA signature predicts survival in stomach adenocarcinoma

Supplementary Materials

Supplementary Table 1: The differentially expressed miRNAs in paired STAD and normal tissues. See Supplementary_Table_1

Supplementary Table 2: The relationship of 8-miRNA signature and clinical parameters

Variable	High risk	Low risk	*P*
Age(yeras)			0.288
< 60	54 (45.0%)	66 (55.0%)	
≥ 60	102 (39.2%)	158 (60.8%)	
Sex			0.588
Male	101(40.1%)	151 (59.9%)	
Female	55 (43.0%)	73 (57.0%)	

Supplementary Table 3: The over-representation analysis for target genes. See Supplementary_Table_3
