# Supplementary material for: A novel microRNA signature predicts survival in stomach adenocarcinoma
Source: Oncotarget. 2017 Mar 7;8(17):28144–53. doi: 10.18632/oncotarget.15961 (PMC5438638; doi:10.18632/oncotarget.15961)
Supplement: Supplementary file 2 [file oncotarget-08-28144-s002.docx]

**Supplementary Table S1. The differentially expressed miRNAs in paired STAD and normal tissues.**

| **miRNA** | **logFC*** | **logCPM*** | **LR*** | **P Value** | **FDR** |
| --- | --- | --- | --- | --- | --- |
| hsa-mir-1269 | 5.24766 | 4.896873 | 153.8731 | 2.47E-35 | 1.24E-33 |
| hsa-mir-196a-1 | 5.173649 | 6.457087 | 393.0208 | 1.82E-87 | 8.23E-85 |
| hsa-mir-196b | 4.826419 | 7.887649 | 348.1235 | 1.09E-77 | 2.45E-75 |
| hsa-mir-548f-1 | 4.040168 | -0.00399 | 67.67345 | 1.93E-16 | 5.13E-15 |
| hsa-mir-196a-2 | 3.993711 | 2.400791 | 162.9158 | 2.61E-37 | 1.47E-35 |
| hsa-mir-552 | 3.718047 | 4.015132 | 117.9429 | 1.78E-27 | 6.72E-26 |
| hsa-mir-135b | 3.186272 | 4.944869 | 171.6598 | 3.21E-39 | 2.07E-37 |
| hsa-mir-549 | 2.68146 | -0.48982 | 35.57181 | 2.46E-09 | 2.22E-08 |
| hsa-mir-509-1 | 2.430465 | -0.2549 | 32.75796 | 1.04E-08 | 8.28E-08 |
| hsa-mir-3687 | 2.324626 | 0.222873 | 38.23519 | 6.27E-10 | 6.30E-09 |
| hsa-mir-615 | 2.176016 | 1.402284 | 53.00359 | 3.33E-13 | 5.79E-12 |
| hsa-mir-105-1 | 2.170972 | 3.821292 | 29.73005 | 4.97E-08 | 3.37E-07 |
| hsa-mir-3176 | 2.160355 | -0.58701 | 26.15449 | 3.15E-07 | 1.74E-06 |
| hsa-mir-1911 | 2.127434 | 0.854113 | 20.84261 | 4.99E-06 | 2.09E-05 |
| hsa-mir-592 | 2.046905 | 1.778939 | 55.78928 | 8.07E-14 | 1.46E-12 |
| hsa-mir-183 | 1.943658 | 11.47269 | 47.08264 | 6.81E-12 | 9.92E-11 |
| hsa-mir-3662 | 1.887278 | -0.58133 | 20.02632 | 7.64E-06 | 3.11E-05 |
| hsa-mir-194-2 | 1.858527 | 12.10746 | 32.49045 | 1.20E-08 | 9.33E-08 |
| hsa-mir-194-1 | 1.827376 | 11.98643 | 31.70942 | 1.79E-08 | 1.35E-07 |
| hsa-mir-3648 | 1.822511 | 1.564576 | 39.0819 | 4.06E-10 | 4.21E-09 |
| hsa-mir-767 | 1.813376 | 3.790524 | 23.19221 | 1.47E-06 | 6.69E-06 |
| hsa-mir-18a | 1.797128 | 4.26354 | 64.10104 | 1.18E-15 | 2.67E-14 |
| hsa-mir-96 | 1.771828 | 3.458154 | 50.32947 | 1.30E-12 | 2.10E-11 |
| hsa-mir-105-2 | 1.771443 | 3.833691 | 19.13901 | 1.22E-05 | 4.74E-05 |
| hsa-mir-192 | 1.719582 | 14.41716 | 22.35297 | 2.27E-06 | 1.01E-05 |
| hsa-mir-200a | 1.69807 | 10.43228 | 36.13127 | 1.84E-09 | 1.74E-08 |
| hsa-mir-215 | 1.685801 | 10.59604 | 23.36748 | 1.34E-06 | 6.24E-06 |
| hsa-mir-551a | 1.646534 | -0.21254 | 18.34646 | 1.84E-05 | 6.94E-05 |
| hsa-mir-1254 | 1.594923 | -0.15366 | 21.86838 | 2.92E-06 | 1.25E-05 |
| hsa-mir-188 | 1.584507 | 2.062049 | 41.67226 | 1.08E-10 | 1.28E-09 |
| hsa-mir-146b | 1.571159 | 8.577653 | 50.55111 | 1.16E-12 | 1.94E-11 |
| hsa-mir-3651 | 1.56792 | 0.552446 | 23.31423 | 1.38E-06 | 6.35E-06 |
| hsa-mir-937 | 1.566361 | 0.824561 | 28.12699 | 1.14E-07 | 6.99E-07 |
| hsa-mir-7-3 | 1.551694 | 0.962984 | 28.57573 | 9.01E-08 | 5.74E-07 |
| hsa-mir-429 | 1.550224 | 8.159935 | 35.86425 | 2.12E-09 | 1.95E-08 |
| hsa-mir-21 | 1.524249 | 17.07657 | 28.22467 | 1.08E-07 | 6.78E-07 |
| hsa-mir-301b | 1.517523 | 0.565191 | 24.26225 | 8.41E-07 | 4.04E-06 |
| hsa-mir-200b | 1.515505 | 10.36933 | 28.65058 | 8.67E-08 | 5.60E-07 |
| hsa-mir-877 | 1.490746 | 1.090363 | 30.53817 | 3.27E-08 | 2.31E-07 |
| hsa-mir-935 | 1.490044 | 1.558525 | 25.22209 | 5.11E-07 | 2.59E-06 |
| hsa-mir-509-2 | 1.484827 | -0.22201 | 13.70959 | 0.000213362 | 0.000626232 |
| hsa-mir-182 | 1.483654 | 12.73574 | 25.78177 | 3.82E-07 | 2.03E-06 |
| hsa-mir-141 | 1.461218 | 10.07376 | 27.45664 | 1.61E-07 | 9.31E-07 |
| hsa-mir-184 | 1.434392 | 3.704775 | 15.82452 | 6.95E-05 | 0.000236182 |
| hsa-mir-509-3 | 1.375675 | -0.11822 | 12.50652 | 0.000405535 | 0.001152841 |
| hsa-mir-1292 | 1.372709 | -0.40602 | 14.58476 | 0.000133994 | 0.000432608 |
| hsa-mir-501 | 1.364548 | 4.953196 | 42.87447 | 5.84E-11 | 7.33E-10 |
| hsa-mir-3944 | 1.359005 | -0.58497 | 12.04175 | 0.000520222 | 0.001451483 |
| hsa-mir-503 | 1.339988 | 2.37236 | 28.07584 | 1.17E-07 | 7.03E-07 |
| hsa-mir-130b | 1.334593 | 4.434231 | 34.33792 | 4.63E-09 | 3.88E-08 |
| hsa-mir-1266 | 1.31746 | 3.625128 | 30.40361 | 3.51E-08 | 2.44E-07 |
| hsa-mir-639 | 1.309998 | -0.29025 | 14.34584 | 0.000152115 | 0.000477473 |
| hsa-mir-577 | 1.286575 | 4.265989 | 23.98078 | 9.73E-07 | 4.63E-06 |
| hsa-mir-1228 | 1.285879 | 0.054366 | 15.80262 | 7.03E-05 | 0.000237149 |
| hsa-mir-556 | 1.284855 | 0.331394 | 16.9532 | 3.83E-05 | 0.000134243 |
| hsa-mir-147b | 1.268672 | 1.916673 | 17.66322 | 2.64E-05 | 9.53E-05 |
| hsa-mir-142 | 1.26474 | 11.22242 | 21.86826 | 2.92E-06 | 1.25E-05 |
| hsa-mir-940 | 1.252802 | 1.526729 | 23.16899 | 1.48E-06 | 6.69E-06 |
| hsa-mir-3194 | 1.245561 | -0.22182 | 12.65948 | 0.000373666 | 0.001068969 |
| hsa-mir-3677 | 1.233403 | 2.618829 | 28.11401 | 1.14E-07 | 6.99E-07 |
| hsa-mir-550a-1 | 1.230369 | 1.765377 | 24.84935 | 6.20E-07 | 3.05E-06 |
| hsa-mir-222 | 1.229276 | 6.354788 | 34.67439 | 3.90E-09 | 3.32E-08 |
| hsa-mir-3127 | 1.205502 | 1.919227 | 25.61662 | 4.16E-07 | 2.19E-06 |
| hsa-mir-335 | 1.200402 | 5.660871 | 34.92476 | 3.43E-09 | 2.98E-08 |
| hsa-mir-1537 | 1.196737 | -0.57095 | 8.840801 | 0.002945699 | 0.006863175 |
| hsa-mir-4326 | 1.178167 | 2.60476 | 22.28298 | 2.35E-06 | 1.03E-05 |
| hsa-mir-19a | 1.169756 | 4.440135 | 29.28147 | 6.26E-08 | 4.16E-07 |
| hsa-mir-1304 | 1.158205 | 0.530508 | 14.85341 | 0.000116198 | 0.000377851 |
| hsa-mir-3682 | 1.133584 | 0.095028 | 13.76865 | 0.000206758 | 0.000610814 |
| hsa-mir-181b-2 | 1.131267 | 1.465516 | 18.29105 | 1.90E-05 | 7.08E-05 |
| hsa-mir-200c | 1.123707 | 12.62303 | 13.92546 | 0.000190205 | 0.000573151 |
| hsa-mir-33b | 1.06251 | 2.191573 | 17.20339 | 3.36E-05 | 0.000118592 |
| hsa-mir-3690 | 1.019013 | -0.45859 | 7.58355 | 0.005890333 | 0.012157217 |
| hsa-mir-19b-1 | 1.017658 | 2.760713 | 19.80815 | 8.56E-06 | 3.46E-05 |
| hsa-mir-1301 | 1.013281 | 2.662935 | 19.28604 | 1.13E-05 | 4.42E-05 |
| hsa-mir-500b | 1.01082 | 2.853586 | 19.51406 | 9.99E-06 | 3.96E-05 |
| hsa-mir-579 | 1.003594 | 0.173085 | 10.04165 | 0.001530405 | 0.003864485 |
| hsa-mir-3199-2 | -1.01231 | 0.049924 | 11.01296 | 0.000904771 | 0.002419862 |
| hsa-mir-30c-2 | -1.01405 | 9.079368 | 21.95033 | 2.80E-06 | 1.22E-05 |
| hsa-mir-381 | -1.04833 | 5.898908 | 27.794 | 1.35E-07 | 7.92E-07 |
| hsa-mir-497 | -1.05245 | 4.887338 | 25.87917 | 3.63E-07 | 1.96E-06 |
| hsa-mir-193a | -1.06783 | 7.615196 | 26.51488 | 2.62E-07 | 1.46E-06 |
| hsa-mir-3678 | -1.06929 | -0.46024 | 9.381958 | 0.002191314 | 0.005296652 |
| hsa-mir-328 | -1.08873 | 4.505851 | 25.32157 | 4.85E-07 | 2.49E-06 |
| hsa-mir-125a | -1.12163 | 8.957969 | 26.09095 | 3.26E-07 | 1.77E-06 |
| hsa-mir-218-1 | -1.13081 | 0.310216 | 12.81953 | 0.000343021 | 0.001000292 |
| hsa-mir-23b | -1.14816 | 10.70065 | 23.39329 | 1.32E-06 | 6.22E-06 |
| hsa-mir-28 | -1.16295 | 12.74368 | 20.38194 | 6.34E-06 | 2.61E-05 |
| hsa-mir-1224 | -1.1755 | 4.110555 | 13.78066 | 0.00020544 | 0.000610814 |
| hsa-mir-1262 | -1.19894 | 0.628874 | 18.60011 | 1.61E-05 | 6.12E-05 |
| hsa-mir-125b-2 | -1.19928 | 4.172908 | 25.12644 | 5.37E-07 | 2.70E-06 |
| hsa-mir-125b-1 | -1.22699 | 9.332394 | 25.33243 | 4.83E-07 | 2.49E-06 |
| hsa-mir-101-2 | -1.238 | 5.831003 | 37.49146 | 9.18E-10 | 8.83E-09 |
| hsa-mir-802 | -1.30525 | 2.120566 | 8.819754 | 0.002979869 | 0.006907183 |
| hsa-mir-1247 | -1.31901 | 4.444355 | 32.06739 | 1.49E-08 | 1.14E-07 |
| hsa-mir-504 | -1.34318 | 0.518477 | 21.00924 | 4.57E-06 | 1.93E-05 |
| hsa-mir-378c | -1.34841 | 3.995761 | 37.56583 | 8.84E-10 | 8.68E-09 |
| hsa-mir-378 | -1.3524 | 9.977656 | 33.84618 | 5.96E-09 | 4.90E-08 |
| hsa-let-7c | -1.37102 | 11.10562 | 25.06968 | 5.53E-07 | 2.75E-06 |
| hsa-mir-195 | -1.38402 | 6.196652 | 46.34211 | 9.93E-12 | 1.36E-10 |
| hsa-mir-365-2 | -1.4117 | 4.593021 | 44.94708 | 2.02E-11 | 2.69E-10 |
| hsa-mir-451 | -1.41848 | 9.482372 | 31.30019 | 2.21E-08 | 1.63E-07 |
| hsa-mir-218-2 | -1.43116 | 5.544884 | 44.19166 | 2.98E-11 | 3.85E-10 |
| hsa-mir-29c | -1.45217 | 12.17281 | 31.27397 | 2.24E-08 | 1.63E-07 |
| hsa-mir-365-1 | -1.45572 | 4.578685 | 47.35135 | 5.93E-12 | 8.94E-11 |
| hsa-mir-20b | -1.4901 | 4.066006 | 39.32045 | 3.60E-10 | 3.96E-09 |
| hsa-mir-551b | -1.50979 | 2.030806 | 29.24939 | 6.36E-08 | 4.17E-07 |
| hsa-mir-100 | -1.5395 | 12.16728 | 30.5749 | 3.21E-08 | 2.30E-07 |
| hsa-mir-99a | -1.53989 | 9.092692 | 39.06369 | 4.10E-10 | 4.21E-09 |
| hsa-mir-149 | -1.56676 | 4.789035 | 49.14688 | 2.37E-12 | 3.70E-11 |
| hsa-mir-202 | -1.60021 | 0.505004 | 26.81042 | 2.24E-07 | 1.28E-06 |
| hsa-mir-363 | -1.6186 | 3.370372 | 46.60951 | 8.66E-12 | 1.22E-10 |
| hsa-mir-144 | -1.6373 | 7.165103 | 56.44918 | 5.77E-14 | 1.09E-12 |
| hsa-mir-30a | -1.65228 | 14.39009 | 35.24595 | 2.91E-09 | 2.58E-08 |
| hsa-mir-23c | -1.65737 | 0.656976 | 33.62034 | 6.70E-09 | 5.41E-08 |
| hsa-mir-605 | -1.70801 | -0.18536 | 24.60363 | 7.04E-07 | 3.42E-06 |
| hsa-mir-206 | -1.79548 | 0.553828 | 28.00989 | 1.21E-07 | 7.18E-07 |
| hsa-mir-143 | -1.8152 | 19.02113 | 26.76652 | 2.30E-07 | 1.30E-06 |
| hsa-mir-9-1 | -1.84622 | 8.838711 | 57.91596 | 2.74E-14 | 5.38E-13 |
| hsa-mir-9-2 | -1.86005 | 8.839239 | 58.76241 | 1.78E-14 | 3.66E-13 |
| hsa-mir-1258 | -1.91162 | 1.044307 | 41.40072 | 1.24E-10 | 1.44E-09 |
| hsa-mir-486 | -1.98119 | 7.630246 | 78.75621 | 7.03E-19 | 2.12E-17 |
| hsa-mir-885 | -1.99205 | -0.00875 | 29.71627 | 5.00E-08 | 3.37E-07 |
| hsa-mir-129-2 | -2.0211 | 3.623788 | 64.84779 | 8.09E-16 | 1.92E-14 |
| hsa-mir-9-3 | -2.10139 | 0.276495 | 41.2004 | 1.37E-10 | 1.55E-09 |
| hsa-mir-187 | -2.15428 | 3.835279 | 61.5645 | 4.28E-15 | 9.22E-14 |
| hsa-mir-137 | -2.17364 | 0.549852 | 39.20199 | 3.82E-10 | 4.11E-09 |
| hsa-mir-129-1 | -2.37581 | 3.5062 | 86.67324 | 1.28E-20 | 4.13E-19 |
| hsa-mir-145 | -2.43625 | 13.4502 | 68.19737 | 1.48E-16 | 4.18E-15 |
| hsa-mir-204 | -2.51743 | 3.768183 | 101.6399 | 6.66E-24 | 2.32E-22 |
| hsa-mir-1-1 | -2.5258 | -0.29066 | 42.01943 | 9.04E-11 | 1.10E-09 |
| hsa-mir-139 | -2.56445 | 6.924574 | 144.909 | 2.25E-33 | 1.02E-31 |
| hsa-mir-383 | -2.58313 | 1.372154 | 65.92232 | 4.69E-16 | 1.18E-14 |
| hsa-mir-133a-2 | -3.28029 | 2.878416 | 142.2048 | 8.77E-33 | 3.60E-31 |
| hsa-mir-133b | -3.43591 | 4.882301 | 180.3691 | 4.03E-41 | 3.64E-39 |
| hsa-mir-133a-1 | -3.47009 | 7.686724 | 189.3515 | 4.40E-43 | 4.98E-41 |
| hsa-mir-1-2 | -3.52462 | 8.462458 | 179.2811 | 6.96E-41 | 5.24E-39 |
| hsa-mir-490 | -4.8051 | 5.883648 | 227.3672 | 2.24E-51 | 3.37E-49 |

*FC: fold change; CPM: Counts Per Million; LR: likehood Raito
