# Supplementary material for: A novel microRNA signature predicts survival in stomach adenocarcinoma
Source: Oncotarget. 2017 Mar 7;8(17):28144–53. doi: 10.18632/oncotarget.15961 (PMC5438638; doi:10.18632/oncotarget.15961)
Supplement: Supplementary file 3 [file oncotarget-08-28144-s003.docx]

**Supplementary Table S3. The over-representation analysis for target genes**

| **Category** | **Subcategory** | **expected** | **observed** | **p-value**  (fdr) |
| --- | --- | --- | --- | --- |
| KEGG | Olfactory transduction | 65.9708 | 4 | 8.70062e-25 |
| KEGG | Pathways in cancer | 55.7691 | 104 | 9.77504e-10 |
| KEGG | Axon guidance | 21.9336 | 48 | 1.29111e-06 |
| KEGG | Systemic lupus erythematosus | 23.9739 | 4 | 4.34052e-06 |
| KEGG | Prostate cancer | 15.1325 | 35 | 1.38788e-05 |
| KEGG | Endocytosis | 34.8557 | 61 | 8.7673e-05 |
| KEGG | Wnt signaling pathway | 25.6742 | 48 | 0.000120727 |
| KEGG | Pancreatic cancer | 11.9019 | 27 | 0.000285899 |
| KEGG | Metabolic pathways | 190.431 | 145 | 0.000393994 |
| KEGG | Focal adhesion | 34.1756 | 57 | 0.00048759 |
| KEGG | Neurotrophin signaling pathway | 21.4235 | 40 | 0.00048759 |
| KEGG | Oxidative phosphorylation | 22.7837 | 7 | 0.00048759 |
| KEGG | MAPK signaling pathway | 45.3974 | 71 | 0.000491469 |
| KEGG | Melanoma | 12.072 | 26 | 0.000679779 |
| KEGG | Small cell lung cancer | 14.2823 | 29 | 0.000838952 |
| KEGG | Chronic myeloid leukemia | 12.412 | 26 | 0.000910191 |
| KEGG | Glioma | 11.0518 | 24 | 0.000910191 |
| KEGG | TGF-beta signaling pathway | 14.4524 | 29 | 0.000910191 |
| KEGG | Renal cell carcinoma | 11.9019 | 25 | 0.00111486 |
| KEGG | Adherens junction | 12.7521 | 25 | 0.00364966 |
| KEGG | Parkinson's disease | 22.4437 | 9 | 0.00367225 |
| KEGG | Thyroid cancer | 4.9308 | 13 | 0.00367225 |
| KEGG | Bacterial invasion of epithelial cells | 12.412 | 24 | 0.00495738 |
| KEGG | Tight junction | 22.7837 | 38 | 0.00495738 |
| KEGG | Drug metabolism - cytochrome P450 | 12.412 | 3 | 0.0056021 |
| KEGG | Bladder cancer | 7.14117 | 16 | 0.00609779 |
| KEGG | Non-small cell lung cancer | 9.1815 | 19 | 0.00626881 |
| KEGG | Metabolism of xenobiotics by cytochrome P450 | 12.072 | 3 | 0.00674252 |
| KEGG | Ribosome | 14.9624 | 5 | 0.00857427 |
| KEGG | Endometrial cancer | 8.84144 | 18 | 0.00887364 |
| KEGG | Regulation of actin cytoskeleton | 36.726 | 54 | 0.00887364 |
| KEGG | mTOR signaling pathway | 8.84144 | 18 | 0.00887364 |
| KEGG | ErbB signaling pathway | 14.7924 | 26 | 0.0107347 |
| KEGG | Basal cell carcinoma | 9.35153 | 18 | 0.0169916 |
| KEGG | Drug metabolism - other enzymes | 8.84144 | 2 | 0.0209838 |
| KEGG | Spliceosome | 21.7636 | 11 | 0.0229902 |
| KEGG | Melanogenesis | 17.3428 | 28 | 0.0254281 |
| KEGG | Colorectal cancer | 10.5417 | 19 | 0.0268231 |
| KEGG | Shigellosis | 10.8818 | 19 | 0.0379988 |
| KEGG | Ubiquitin mediated proteolysis | 23.6339 | 35 | 0.0388766 |
| KEGG | p53 signaling pathway | 11.7319 | 20 | 0.0397434 |
| KEGG | Circadian rhythm - mammal | 3.91064 | 9 | 0.041866 |
| KEGG | Apoptosis | 14.9624 | 24 | 0.0425254 |
| KEGG | Long-term potentiation | 11.9019 | 20 | 0.0438062 |
| KEGG | Type I diabetes mellitus | 7.65125 | 2 | 0.0467255 |
| Gene Ontology | protein binding | 1439.31 | 1744 | 1.20773e-29 |
| Gene Ontology | olfactory receptor activity | 74.0326 | 2 | 1.678e-29 |
| Gene Ontology | regulation of biological process | 1161.91 | 1453 | 1.03598e-28 |
| Gene Ontology | regulation of cellular process | 1104.3 | 1391 | 1.4234e-28 |
| Gene Ontology | biological regulation | 1231.7 | 1524 | 1.4234e-28 |
| Gene Ontology | sensory perception of chemical stimulus | 84.4572 | 7 | 2.91703e-28 |
| Gene Ontology | sensory perception of smell | 76.3295 | 4 | 2.91703e-28 |
| Gene Ontology | binding | 2188.11 | 2448 | 2.50247e-27 |
| Gene Ontology | cellular process | 2053.48 | 2305 | 1.66074e-23 |
| Gene Ontology | regulation of macromolecule metabolic process | 602.685 | 800 | 3.28602e-19 |
| Gene Ontology | regulation of gene expression | 522.645 | 709 | 4.89646e-19 |
| Gene Ontology | intracellular | 2001.71 | 2230 | 8.97388e-19 |
| Gene Ontology | nervous system development | 207.433 | 333 | 2.00503e-18 |
| Gene Ontology | anatomical structure development | 504.093 | 683 | 4.09267e-18 |
| Gene Ontology | regulation of cellular biosynthetic process | 538.547 | 721 | 5.81412e-18 |
| Gene Ontology | regulation of macromolecule biosynthetic process | 512.574 | 691 | 7.5782e-18 |
| Gene Ontology | signaling | 630.956 | 823 | 8.10013e-18 |
| Gene Ontology | regulation of metabolic process | 698.097 | 896 | 1.04263e-17 |
| Gene Ontology | developmental process | 604.629 | 793 | 1.04263e-17 |
| Gene Ontology | regulation of biosynthetic process | 542.435 | 723 | 1.28287e-17 |
| Gene Ontology | regulation of primary metabolic process | 633.429 | 824 | 1.384e-17 |
| Gene Ontology | system development | 454.62 | 623 | 1.50242e-17 |
| Gene Ontology | regulation of cellular metabolic process | 665.763 | 858 | 2.48704e-17 |
| Gene Ontology | intracellular part | 1939.34 | 2155 | 1.67357e-16 |
| Gene Ontology | regulation of nitrogen compound metabolic process | 541.728 | 715 | 2.52513e-16 |
| Gene Ontology | regulation of nucleobase, nucleoside, nucleotide and nucleic acid metabolic process | 537.134 | 708 | 5.46062e-16 |
| Gene Ontology | signaling pathway | 450.026 | 608 | 1.18926e-15 |
| Gene Ontology | regulation of transcription | 467.695 | 626 | 2.86616e-15 |
| Gene Ontology | multicellular organismal development | 551.092 | 719 | 3.41987e-15 |
| Gene Ontology | signaling process | 455.327 | 611 | 4.18361e-15 |
| Gene Ontology | signal transmission | 454.267 | 609 | 5.78423e-15 |
| Gene Ontology | transcription | 484.834 | 639 | 3.87935e-14 |
| Gene Ontology | cellular macromolecule metabolic process | 1006.95 | 1199 | 1.21582e-13 |
| Gene Ontology | intracellular signaling pathway | 268.92 | 386 | 3.35321e-13 |
| Gene Ontology | G-protein coupled receptor activity | 151.069 | 72 | 3.46421e-13 |
| Gene Ontology | signal transduction | 394.369 | 530 | 6.41748e-13 |
| Gene Ontology | intracellular signal transduction | 212.91 | 314 | 4.49323e-12 |
| Gene Ontology | sensory perception | 146.652 | 72 | 5.1963e-12 |
| Gene Ontology | organelle | 1653.81 | 1843 | 6.58746e-12 |
| Gene Ontology | intracellular organelle | 1651.33 | 1840 | 7.57579e-12 |
| Gene Ontology | localization | 594.028 | 745 | 9.76882e-12 |
| Gene Ontology | macromolecule localization | 221.921 | 321 | 3.01326e-11 |
| Gene Ontology | anatomical structure morphogenesis | 246.658 | 349 | 5.78114e-11 |
| Gene Ontology | intracellular membrane-bounded organelle | 1479.95 | 1660 | 9.85731e-11 |
| Gene Ontology | membrane-bounded organelle | 1481.18 | 1661 | 1.03187e-10 |
| Gene Ontology | cellular macromolecule biosynthetic process | 623.535 | 768 | 2.05736e-10 |
| Gene Ontology | cell communication | 298.074 | 405 | 3.19911e-10 |
| Gene Ontology | nucleus | 918.428 | 1079 | 4.52229e-10 |
| Gene Ontology | cytoskeletal protein binding | 90.4647 | 153 | 5.05486e-10 |
| Gene Ontology | macromolecule biosynthetic process | 635.55 | 777 | 7.06211e-10 |
| Gene Ontology | small GTPase mediated signal transduction | 76.5062 | 134 | 7.13011e-10 |
| Gene Ontology | transcription regulator activity | 269.097 | 369 | 8.78054e-10 |
| Gene Ontology | organ development | 341.539 | 452 | 8.78054e-10 |
| Gene Ontology | metal ion binding | 668.944 | 812 | 8.81693e-10 |
| Gene Ontology | macromolecule metabolic process | 1108.55 | 1273 | 1.06608e-09 |
| Gene Ontology | positive regulation of cellular process | 360.092 | 472 | 1.23507e-09 |
| Gene Ontology | regulation of cell communication | 207.256 | 295 | 1.68966e-09 |
| Gene Ontology | ion binding | 677.778 | 819 | 1.78015e-09 |
| Gene Ontology | positive regulation of biological process | 395.783 | 511 | 1.78015e-09 |
| Gene Ontology | cell part | 2661.99 | 2780 | 1.88196e-09 |
| Gene Ontology | cell | 2662.17 | 2780 | 1.9373e-09 |
| Gene Ontology | post-translational protein modification | 266.623 | 364 | 1.9373e-09 |
| Gene Ontology | establishment of localization | 514.164 | 641 | 2.2602e-09 |
| Gene Ontology | protein modification process | 312.386 | 416 | 2.34452e-09 |
| Gene Ontology | regulation of signaling pathway | 180.576 | 262 | 2.70921e-09 |
| Gene Ontology | cation binding | 676.011 | 815 | 3.02337e-09 |
| Gene Ontology | protein localization | 185.17 | 266 | 5.6312e-09 |
| Gene Ontology | vesicle-mediated transport | 118.735 | 185 | 5.67848e-09 |
| Gene Ontology | transport | 507.804 | 631 | 5.77634e-09 |
| Gene Ontology | cytoplasm | 1356.26 | 1517 | 8.7978e-09 |
| Gene Ontology | cellular developmental process | 339.949 | 443 | 1.13918e-08 |
| Gene Ontology | regulation of RNA metabolic process | 330.231 | 432 | 1.14156e-08 |
| Gene Ontology | neuron projection | 65.5515 | 115 | 1.39421e-08 |
| Gene Ontology | enzyme linked receptor protein signaling pathway | 83.9272 | 139 | 1.60159e-08 |
| Gene Ontology | plasma membrane part | 357.441 | 461 | 2.00991e-08 |
| Gene Ontology | positive regulation of nitrogen compound metabolic process | 123.859 | 189 | 2.07766e-08 |
| Gene Ontology | cell junction | 95.2352 | 153 | 2.19298e-08 |
| Gene Ontology | transcription factor activity | 170.328 | 245 | 2.52347e-08 |
| Gene Ontology | transmembrane receptor activity | 225.278 | 148 | 2.95304e-08 |
| Gene Ontology | macromolecule modification | 326.521 | 425 | 3.04106e-08 |
| Gene Ontology | negative regulation of cellular process | 333.235 | 432 | 3.70751e-08 |
| Gene Ontology | cellular component organization | 496.142 | 612 | 3.81962e-08 |
| Gene Ontology | positive regulation of nucleobase, nucleoside, nucleotide and nucleic acid metabolic process | 120.148 | 183 | 4.30999e-08 |
| Gene Ontology | establishment of protein localization | 159.727 | 231 | 4.55279e-08 |
| Gene Ontology | cellular biosynthetic process | 764.356 | 898 | 5.62399e-08 |
| Gene Ontology | cell differentiation | 324.047 | 420 | 6.54167e-08 |
| Gene Ontology | neuron development | 70.4988 | 119 | 7.84883e-08 |
| Gene Ontology | gene expression | 672.477 | 799 | 8.43778e-08 |
| Gene Ontology | protein amino acid phosphorylation | 142.588 | 209 | 8.72931e-08 |
| Gene Ontology | Ras protein signal transduction | 40.4617 | 78 | 8.98756e-08 |
| Gene Ontology | cellular metabolic process | 1312.97 | 1462 | 1.06844e-07 |
| Gene Ontology | intracellular transport | 133.047 | 197 | 1.06844e-07 |
| Gene Ontology | negative regulation of biological process | 364.686 | 463 | 1.45621e-07 |
| Gene Ontology | cellular localization | 213.087 | 291 | 1.57503e-07 |
| Gene Ontology | cell morphogenesis | 75.9762 | 125 | 1.58289e-07 |
| Gene Ontology | regulation of transcription, DNA-dependent | 321.574 | 414 | 1.93164e-07 |
| Gene Ontology | biosynthetic process | 783.791 | 912 | 2.74632e-07 |
| Gene Ontology | positive regulation of gene expression | 111.667 | 169 | 2.74632e-07 |
| Gene Ontology | nucleic acid metabolic process | 657.989 | 779 | 2.74632e-07 |
| Gene Ontology | zinc ion binding | 354.968 | 450 | 2.91415e-07 |
| Gene Ontology | protein transport | 156.9 | 223 | 3.77259e-07 |
| Gene Ontology | Golgi apparatus | 157.783 | 224 | 3.82629e-07 |
| Gene Ontology | establishment of localization in cell | 197.185 | 270 | 4.32623e-07 |
| Gene Ontology | cognition | 163.967 | 103 | 4.58074e-07 |
| Gene Ontology | transition metal ion binding | 403.734 | 502 | 5.17671e-07 |
| Gene Ontology | generation of neurons | 106.19 | 161 | 5.17671e-07 |
| Gene Ontology | positive regulation of metabolic process | 185.877 | 256 | 5.98561e-07 |
| Gene Ontology | nucleobase, nucleoside, nucleotide and nucleic acid metabolic process | 758.702 | 882 | 6.40271e-07 |
| Gene Ontology | cell projection | 135.344 | 196 | 6.48035e-07 |
| Gene Ontology | cellular macromolecule localization | 88.8745 | 139 | 6.52034e-07 |
| Gene Ontology | cell development | 142.058 | 204 | 6.52099e-07 |
| Gene Ontology | molecular_function | 2731.96 | 2825 | 7.40513e-07 |
| Gene Ontology | neurogenesis | 114.141 | 170 | 7.40513e-07 |
| Gene Ontology | neuron differentiation | 96.4721 | 148 | 8.42314e-07 |
| Gene Ontology | cellular protein localization | 88.5211 | 138 | 8.74799e-07 |
| Gene Ontology | extracellular region | 359.738 | 273 | 9.6376e-07 |
| Gene Ontology | positive regulation of macromolecule metabolic process | 172.448 | 239 | 9.76744e-07 |
| Gene Ontology | positive regulation of transcription | 105.837 | 159 | 1.12115e-06 |
| Gene Ontology | cellular membrane organization | 78.6265 | 125 | 1.17046e-06 |
| Gene Ontology | membrane organization | 78.8032 | 125 | 1.33674e-06 |
| Gene Ontology | primary metabolic process | 1347.96 | 1485 | 1.41491e-06 |
| Gene Ontology | intracellular protein transport | 77.5664 | 123 | 1.72703e-06 |
| Gene Ontology | positive regulation of transcription, DNA-dependent | 91.1714 | 140 | 1.78254e-06 |
| Gene Ontology | transmembrane receptor protein tyrosine kinase signaling pathway | 52.2999 | 90 | 2.09046e-06 |
| Gene Ontology | positive regulation of RNA metabolic process | 92.2315 | 141 | 2.10859e-06 |
| Gene Ontology | cell death | 220.861 | 293 | 2.12041e-06 |
| Gene Ontology | neuron projection development | 56.8938 | 96 | 2.13818e-06 |
| Gene Ontology | regulation of transcription from RNA polymerase II promoter | 134.283 | 192 | 2.15744e-06 |
| Gene Ontology | negative regulation of gene expression | 101.243 | 152 | 2.17145e-06 |
| Gene Ontology | positive regulation of cellular biosynthetic process | 131.81 | 189 | 2.17145e-06 |
| Gene Ontology | regulation of signal transduction | 139.407 | 198 | 2.21864e-06 |
| Gene Ontology | regulation of signaling process | 140.291 | 199 | 2.24342e-06 |
| Gene Ontology | cellular component morphogenesis | 83.5738 | 130 | 2.24342e-06 |
| Gene Ontology | transcription, DNA-dependent | 344.366 | 431 | 2.5032e-06 |
| Gene Ontology | death | 221.391 | 293 | 2.51351e-06 |
| Gene Ontology | positive regulation of cellular metabolic process | 176.512 | 241 | 2.82212e-06 |
| Gene Ontology | RNA biosynthetic process | 345.073 | 431 | 3.15743e-06 |
| Gene Ontology | positive regulation of biosynthetic process | 133.753 | 190 | 3.87486e-06 |
| Gene Ontology | negative regulation of macromolecule biosynthetic process | 107.957 | 159 | 3.87486e-06 |
| Gene Ontology | biological_process | 2529.83 | 2633 | 4.2933e-06 |
| Gene Ontology | synapse | 65.5515 | 106 | 4.44597e-06 |
| Gene Ontology | regulation of developmental process | 142.588 | 200 | 4.7308e-06 |
| Gene Ontology | regulation of cellular component organization | 97.7089 | 146 | 4.93779e-06 |
| Gene Ontology | posttranscriptional regulation of gene expression | 43.6421 | 77 | 5.3146e-06 |
| Gene Ontology | DNA binding | 414.335 | 505 | 5.4104e-06 |
| Gene Ontology | programmed cell death | 201.602 | 268 | 5.90722e-06 |
| Gene Ontology | apoptosis | 200.188 | 266 | 6.82307e-06 |
| Gene Ontology | positive regulation of transcription from RNA polymerase II promoter | 70.3221 | 111 | 8.38302e-06 |
| Gene Ontology | negative regulation of transcription | 90.4647 | 136 | 8.75352e-06 |
| Gene Ontology | tube development | 60.2509 | 98 | 9.20521e-06 |
| Gene Ontology | negative regulation of cellular biosynthetic process | 111.137 | 161 | 9.27006e-06 |
| Gene Ontology | cellular protein metabolic process | 435.538 | 526 | 9.29184e-06 |
| Gene Ontology | cell projection organization | 80.9235 | 124 | 9.60363e-06 |
| Gene Ontology | negative regulation of biosynthetic process | 112.904 | 163 | 9.67715e-06 |
| Gene Ontology | cellular nitrogen compound metabolic process | 809.941 | 923 | 9.67715e-06 |
| Gene Ontology | intracellular protein kinase cascade | 105.483 | 154 | 9.7113e-06 |
| Gene Ontology | signal transmission via phosphorylation event | 105.483 | 154 | 9.7113e-06 |
| Gene Ontology | anatomical structure formation involved in morphogenesis | 83.3971 | 127 | 9.7113e-06 |
| Gene Ontology | organ morphogenesis | 116.438 | 167 | 1.05512e-05 |
| Gene Ontology | regulation of cell death | 159.727 | 218 | 1.06916e-05 |
| Gene Ontology | positive regulation of macromolecule biosynthetic process | 124.036 | 176 | 1.07611e-05 |
| Gene Ontology | morphogenesis of an epithelium | 39.4016 | 70 | 1.28336e-05 |
| Gene Ontology | regulation of anatomical structure morphogenesis | 55.3036 | 91 | 1.28336e-05 |
| Gene Ontology | protein localization in organelle | 37.2813 | 67 | 1.39804e-05 |
| Gene Ontology | cell morphogenesis involved in differentiation | 53.8901 | 89 | 1.42236e-05 |
| Gene Ontology | transcription from RNA polymerase II promoter | 162.377 | 220 | 1.62883e-05 |
| Gene Ontology | response to oxygen levels | 28.0935 | 54 | 1.70327e-05 |
| Gene Ontology | nitrogen compound metabolic process | 830.084 | 941 | 1.74826e-05 |
| Gene Ontology | enzyme binding | 113.258 | 162 | 1.78623e-05 |
| Gene Ontology | muscle structure development | 54.2435 | 89 | 1.88464e-05 |
| Gene Ontology | heart development | 42.9354 | 74 | 2.14295e-05 |
| Gene Ontology | protein import into nucleus | 24.2064 | 48 | 2.49732e-05 |
| Gene Ontology | phosphorus metabolic process | 231.109 | 297 | 2.6729e-05 |
| Gene Ontology | phosphate metabolic process | 231.109 | 297 | 2.6729e-05 |
| Gene Ontology | regulation of localization | 130.926 | 182 | 2.69666e-05 |
| Gene Ontology | regulation of programmed cell death | 158.49 | 214 | 2.86911e-05 |
| Gene Ontology | endocytosis | 48.7661 | 81 | 3.32579e-05 |
| Gene Ontology | membrane invagination | 48.7661 | 81 | 3.32579e-05 |
| Gene Ontology | nucleotide binding | 398.787 | 481 | 3.38054e-05 |
| Gene Ontology | actin binding | 58.1306 | 93 | 3.40793e-05 |
| Gene Ontology | regulation of cell morphogenesis | 28.0935 | 53 | 3.8376e-05 |
| Gene Ontology | neuron projection morphogenesis | 43.6421 | 74 | 4.00076e-05 |
| Gene Ontology | response to organic substance | 159.55 | 214 | 4.56141e-05 |
| Gene Ontology | intermediate filament | 31.4506 | 10 | 4.73565e-05 |
| Gene Ontology | regulation of apoptosis | 157.076 | 211 | 4.73565e-05 |
| Gene Ontology | membrane raft | 26.8567 | 51 | 4.73565e-05 |
| Gene Ontology | nuclear import | 24.7364 | 48 | 4.73565e-05 |
| Gene Ontology | muscle organ development | 45.409 | 76 | 4.83489e-05 |
| Gene Ontology | positive regulation of cell death | 83.5738 | 124 | 4.83489e-05 |
| Gene Ontology | tissue morphogenesis | 51.5931 | 84 | 4.8726e-05 |
| Gene Ontology | response to hypoxia | 26.3266 | 50 | 5.75775e-05 |
| Gene Ontology | basolateral plasma membrane | 39.5783 | 68 | 6.00606e-05 |
| Gene Ontology | beta-catenin binding | 8.12768 | 22 | 6.99081e-05 |
| Gene Ontology | protein localization in nucleus | 27.2101 | 51 | 6.99081e-05 |
| Gene Ontology | protein import | 31.804 | 57 | 9.1285e-05 |
| Gene Ontology | cell surface receptor linked signaling pathway | 264.503 | 330 | 9.89298e-05 |
| Gene Ontology | negative regulation of nucleobase, nucleoside, nucleotide and nucleic acid metabolic process | 101.419 | 144 | 9.98625e-05 |
| Gene Ontology | protein kinase activity | 104.953 | 148 | 0.000109395 |
| Gene Ontology | keratin filament | 15.902 | 2 | 0.000134355 |
| Gene Ontology | response to hormone stimulus | 81.2768 | 119 | 0.000145967 |
| Gene Ontology | intermediate filament cytoskeleton | 33.0408 | 12 | 0.000145967 |
| Gene Ontology | cell-cell junction | 35.3378 | 61 | 0.000158012 |
| Gene Ontology | ribonucleotide binding | 327.051 | 397 | 0.000159132 |
| Gene Ontology | purine ribonucleotide binding | 327.051 | 397 | 0.000159132 |
| Gene Ontology | cell projection morphogenesis | 50.0029 | 80 | 0.00016299 |
| Gene Ontology | response to endogenous stimulus | 90.6413 | 130 | 0.000164511 |
| Gene Ontology | tissue development | 146.122 | 195 | 0.000165589 |
| Gene Ontology | negative regulation of macromolecule metabolic process | 146.122 | 195 | 0.000165589 |
| Gene Ontology | negative regulation of nitrogen compound metabolic process | 102.479 | 144 | 0.000170629 |
| Gene Ontology | endomembrane system | 256.729 | 319 | 0.000195278 |
| Gene Ontology | receptor activity | 298.251 | 235 | 0.000199638 |
| Gene Ontology | positive regulation of programmed cell death | 82.6903 | 120 | 0.000199638 |
| Gene Ontology | Wnt receptor signaling pathway | 33.3942 | 58 | 0.000203528 |
| Gene Ontology | cell leading edge | 29.6837 | 53 | 0.000203528 |
| Gene Ontology | regulation of cell differentiation | 99.4758 | 140 | 0.000203528 |
| Gene Ontology | vesicle organization | 10.4246 | 25 | 0.000203809 |
| Gene Ontology | cellular response to insulin stimulus | 16.2554 | 34 | 0.000206773 |
| Gene Ontology | phosphotransferase activity, alcohol group as acceptor | 125.096 | 170 | 0.000207979 |
| Gene Ontology | cytoskeleton | 249.131 | 310 | 0.000225129 |
| Gene Ontology | blood vessel development | 60.0742 | 92 | 0.000228916 |
| Gene Ontology | cell adhesion | 146.122 | 194 | 0.00023133 |
| Gene Ontology | protein targeting | 48.236 | 77 | 0.000243241 |
| Gene Ontology | brain development | 56.187 | 87 | 0.000246896 |
| Gene Ontology | biological adhesion | 146.298 | 194 | 0.000247059 |
| Gene Ontology | apicolateral plasma membrane | 15.7253 | 33 | 0.000247227 |
| Gene Ontology | purine nucleotide binding | 341.716 | 411 | 0.000247227 |
| Gene Ontology | epithelium development | 65.9049 | 99 | 0.000250098 |
| Gene Ontology | negative regulation of cellular metabolic process | 142.941 | 190 | 0.000256879 |
| Gene Ontology | cellular response to peptide hormone stimulus | 17.8456 | 36 | 0.000276864 |
| Gene Ontology | tube morphogenesis | 44.5256 | 72 | 0.000277875 |
| Gene Ontology | response to insulin stimulus | 24.2064 | 45 | 0.000282884 |
| Gene Ontology | central nervous system development | 81.8069 | 118 | 0.000291564 |
| Gene Ontology | RNA metabolic process | 458.684 | 536 | 0.00029601 |
| Gene Ontology | phosphorylation | 201.602 | 256 | 0.000299056 |
| Gene Ontology | growth | 95.4119 | 134 | 0.000322958 |
| Gene Ontology | response to peptide hormone stimulus | 35.5144 | 60 | 0.000334865 |
| Gene Ontology | negative regulation of transcription, DNA-dependent | 72.9725 | 107 | 0.00033723 |
| Gene Ontology | positive regulation of apoptosis | 82.1603 | 118 | 0.00035102 |
| Gene Ontology | metabolic process | 1480.83 | 1587 | 0.00036127 |
| Gene Ontology | negative regulation of mitotic cell cycle | 3.88715 | 13 | 0.00036127 |
| Gene Ontology | MAPKKK cascade | 52.8299 | 82 | 0.000367951 |
| Gene Ontology | cell morphogenesis involved in neuron differentiation | 42.582 | 69 | 0.000368732 |
| Gene Ontology | negative regulation of metabolic process | 157.076 | 205 | 0.000375149 |
| Gene Ontology | muscle tissue development | 30.3905 | 53 | 0.0003774 |
| Gene Ontology | striated muscle tissue development | 28.977 | 51 | 0.000407091 |
| Gene Ontology | negative regulation of RNA metabolic process | 74.2093 | 108 | 0.000420423 |
| Gene Ontology | Rho protein signal transduction | 21.7327 | 41 | 0.00042755 |
| Gene Ontology | tube formation | 10.2479 | 24 | 0.000433844 |
| Gene Ontology | regulation of biological quality | 304.435 | 368 | 0.000438092 |
| Gene Ontology | nucleoside-triphosphatase regulator activity | 75.9762 | 110 | 0.000442656 |
| Gene Ontology | kinase activity | 135.874 | 180 | 0.000487081 |
| Gene Ontology | cell part morphogenesis | 52.4766 | 81 | 0.000490878 |
| Gene Ontology | axon | 31.4506 | 54 | 0.000496972 |
| Gene Ontology | negative regulation of signal transduction | 21.9094 | 41 | 0.000516282 |
| Gene Ontology | vasculature development | 61.4877 | 92 | 0.000530468 |
| Gene Ontology | structural constituent of ribosome | 27.9168 | 10 | 0.000532387 |
| Gene Ontology | cell migration | 81.6302 | 116 | 0.000632585 |
| Gene Ontology | guanyl-nucleotide exchange factor activity | 26.5033 | 47 | 0.000649358 |
| Gene Ontology | small conjugating protein ligase activity | 37.1046 | 61 | 0.000649358 |
| Gene Ontology | embryonic development | 110.607 | 150 | 0.000654704 |
| Gene Ontology | nucleic acid binding | 579.716 | 660 | 0.000669273 |
| Gene Ontology | protein serine/threonine kinase activity | 75.0927 | 108 | 0.000675429 |
| Gene Ontology | transcription factor binding | 92.7616 | 129 | 0.00067889 |
| Gene Ontology | small GTPase regulator activity | 51.4164 | 79 | 0.000696129 |
| Gene Ontology | multicellular organismal process | 817.892 | 908 | 0.000696129 |
| Gene Ontology | GTPase regulator activity | 74.386 | 107 | 0.000714408 |
| Gene Ontology | regulation of homeostatic process | 23.6763 | 43 | 0.000714408 |
| Gene Ontology | response to chemical stimulus | 267.86 | 326 | 0.00072263 |
| Gene Ontology | anterior/posterior pattern formation | 27.3868 | 48 | 0.0007253 |
| Gene Ontology | transcription repressor activity | 60.4276 | 90 | 0.000734854 |
| Gene Ontology | negative regulation of signaling process | 22.2628 | 41 | 0.000737542 |
| Gene Ontology | regulation of translation | 26.68 | 47 | 0.000745872 |
| Gene Ontology | transcription corepressor activity | 25.9732 | 46 | 0.000771328 |
| Gene Ontology | cellular component movement | 115.378 | 155 | 0.000773994 |
| Gene Ontology | synapse part | 48.4127 | 75 | 0.000777284 |
| Gene Ontology | gland development | 36.0445 | 59 | 0.000932254 |
| Gene Ontology | ubiquitin-protein ligase activity | 33.7476 | 56 | 0.000943855 |
| Gene Ontology | nucleocytoplasmic transport | 39.2249 | 63 | 0.00097846 |
| Gene Ontology | pattern specification process | 50.3563 | 77 | 0.000983976 |
| Gene Ontology | nuclear lumen | 268.214 | 325 | 0.00101892 |
| Gene Ontology | axonogenesis | 38.5182 | 62 | 0.00103092 |
| Gene Ontology | regulation of anatomical structure size | 73.5025 | 105 | 0.00107496 |
| Gene Ontology | cell-cell adhesion | 56.187 | 84 | 0.00108651 |
| Gene Ontology | nuclear transport | 39.4016 | 63 | 0.00110812 |
| Gene Ontology | positive regulation of myeloid cell differentiation | 6.53748 | 17 | 0.0012518 |
| Gene Ontology | regulation of multicellular organismal process | 191.884 | 240 | 0.00130689 |
| Gene Ontology | anchoring junction | 28.8003 | 49 | 0.00132042 |
| Gene Ontology | adherens junction | 25.7966 | 45 | 0.00132529 |
| Gene Ontology | morphogenesis of a branching epithelium | 19.9658 | 37 | 0.0013972 |
| Gene Ontology | epithelial tube morphogenesis | 24.3831 | 43 | 0.00142559 |
| Gene Ontology | perinuclear region of cytoplasm | 58.3073 | 86 | 0.00145605 |
| Gene Ontology | actin cytoskeleton organization | 50.1796 | 76 | 0.00147277 |
| Gene Ontology | regulation of protein metabolic process | 115.201 | 153 | 0.0014819 |
| Gene Ontology | positive regulation of cell communication | 74.2093 | 105 | 0.00154823 |
| Gene Ontology | oxidative phosphorylation | 18.3756 | 5 | 0.00156208 |
| Gene Ontology | positive regulation of signaling pathway | 68.3786 | 98 | 0.00156208 |
| Gene Ontology | transmembrane receptor protein kinase activity | 14.4885 | 29 | 0.00171554 |
| Gene Ontology | apical junction complex | 15.1952 | 30 | 0.00174216 |
| Gene Ontology | nuclear part | 342.423 | 403 | 0.00175957 |
| Gene Ontology | regulation of cellular protein metabolic process | 101.773 | 137 | 0.00176772 |
| Gene Ontology | organelle organization | 265.21 | 319 | 0.0019862 |
| Gene Ontology | tight junction | 13.2517 | 27 | 0.00205031 |
| Gene Ontology | occluding junction | 13.2517 | 27 | 0.00205031 |
| Gene Ontology | glucose import | 6.18411 | 16 | 0.002067 |
| Gene Ontology | actin filament-based process | 53.1833 | 79 | 0.00207236 |
| Gene Ontology | ureteric bud development | 10.6013 | 23 | 0.00214736 |
| Gene Ontology | gland morphogenesis | 15.3719 | 30 | 0.00214961 |
| Gene Ontology | actin filament | 7.42093 | 18 | 0.00217164 |
| Gene Ontology | ribonucleoprotein complex | 89.0511 | 59 | 0.00217164 |
| Gene Ontology | myeloid cell differentiation | 27.9168 | 47 | 0.00224667 |
| Gene Ontology | regulation of cell size | 50.8864 | 76 | 0.00224977 |
| Gene Ontology | rRNA metabolic process | 17.8456 | 5 | 0.00231021 |
| Gene Ontology | epithelial tube formation | 9.3645 | 21 | 0.00236203 |
| Gene Ontology | enzyme regulator activity | 154.426 | 196 | 0.00239361 |
| Gene Ontology | oxidoreductase activity | 121.739 | 87 | 0.00251456 |
| Gene Ontology | regulation of phosphorylation | 93.9984 | 127 | 0.00251456 |
| Gene Ontology | recycling endosome | 6.89086 | 17 | 0.00252459 |
| Gene Ontology | SH3 domain binding | 17.6689 | 33 | 0.00256606 |
| Gene Ontology | protein domain specific binding | 69.4387 | 98 | 0.00267117 |
| Gene Ontology | cytoplasmic part | 915.955 | 1000 | 0.00276342 |
| Gene Ontology | regulation of cellular component size | 62.0178 | 89 | 0.0028323 |
| Gene Ontology | excitatory synapse | 0.883444 | 5 | 0.00287288 |
| Gene Ontology | regulation of small GTPase mediated signal transduction | 48.0593 | 72 | 0.00290052 |
| Gene Ontology | filopodium assembly | 4.06384 | 12 | 0.0029416 |
| Gene Ontology | urogenital system development | 25.9732 | 44 | 0.00296238 |
| Gene Ontology | receptor signaling protein activity | 28.2702 | 47 | 0.00296643 |
| Gene Ontology | ubiquitin protein ligase binding | 12.1915 | 25 | 0.00298562 |
| Gene Ontology | regulation of cell morphogenesis involved in differentiation | 17.8456 | 33 | 0.00307623 |
| Gene Ontology | cell-substrate adhesion | 26.8567 | 45 | 0.00328173 |
| Gene Ontology | neurological system process | 224.748 | 179 | 0.00329628 |
| Gene Ontology | transferase activity, transferring phosphorus-containing groups | 158.136 | 199 | 0.00331376 |
| Gene Ontology | ribosome | 34.9844 | 17 | 0.00331956 |
| Gene Ontology | negative regulation of transcription from RNA polymerase II promoter | 51.5931 | 76 | 0.00335252 |
| Gene Ontology | positive regulation of phosphorus metabolic process | 25.4432 | 43 | 0.00358344 |
| Gene Ontology | positive regulation of phosphate metabolic process | 25.4432 | 43 | 0.00358344 |
| Gene Ontology | purine nucleoside binding | 286.236 | 339 | 0.00364615 |
| Gene Ontology | protein import into nucleus, translocation | 9.01113 | 20 | 0.00370864 |
| Gene Ontology | positive regulation of erythrocyte differentiation | 2.12027 | 8 | 0.00377081 |
| Gene Ontology | rRNA processing | 17.1388 | 5 | 0.00382266 |
| Gene Ontology | regulation of protein amino acid phosphorylation | 40.4617 | 62 | 0.00389386 |
| Gene Ontology | positive regulation of developmental process | 62.7245 | 89 | 0.00402927 |
| Gene Ontology | cell-cell adherens junction | 6.53748 | 16 | 0.0040815 |
| Gene Ontology | regulation of Ras protein signal transduction | 41.3452 | 63 | 0.004086 |
| Gene Ontology | muscle cell differentiation | 25.6199 | 43 | 0.00411411 |
| Gene Ontology | cell motility | 87.4609 | 118 | 0.00411411 |
| Gene Ontology | localization of cell | 87.4609 | 118 | 0.00411411 |
| Gene Ontology | oxidation reduction | 114.318 | 82 | 0.00411928 |
| Gene Ontology | adenyl ribonucleotide binding | 265.563 | 316 | 0.00413273 |
| Gene Ontology | central nervous system neuron development | 4.7706 | 13 | 0.00415062 |
| Gene Ontology | RNA polymerase II transcription factor activity | 42.2286 | 64 | 0.00422773 |
| Gene Ontology | positive regulation of phosphorylation | 24.9131 | 42 | 0.00426393 |
| Gene Ontology | coated pit | 8.48106 | 19 | 0.00438563 |
| Gene Ontology | blood vessel morphogenesis | 52.1232 | 76 | 0.00443449 |
| Gene Ontology | Golgi vesicle transport | 24.2064 | 41 | 0.00445364 |
| Gene Ontology | phospholipid-translocating ATPase activity | 2.65033 | 9 | 0.00449583 |
| Gene Ontology | microspike assembly | 4.24053 | 12 | 0.00449583 |
| Gene Ontology | serine hydrolase activity | 31.6273 | 15 | 0.00451433 |
| Gene Ontology | regionalization | 39.9317 | 61 | 0.00451475 |
| Gene Ontology | non-membrane-bounded organelle | 475.293 | 539 | 0.0045904 |
| Gene Ontology | intracellular non-membrane-bounded organelle | 475.293 | 539 | 0.0045904 |
| Gene Ontology | embryonic epithelial tube formation | 9.18782 | 20 | 0.00468251 |
| Gene Ontology | regulation of phosphate metabolic process | 98.239 | 130 | 0.00468251 |
| Gene Ontology | regulation of phosphorus metabolic process | 98.239 | 130 | 0.00468251 |
| Gene Ontology | peptidyl-lysine modification | 3.1804 | 10 | 0.00473523 |
| Gene Ontology | dendrite | 32.8641 | 52 | 0.00481702 |
| Gene Ontology | induction of apoptosis | 59.014 | 84 | 0.00494307 |
| Gene Ontology | protein metabolic process | 518.582 | 584 | 0.0049586 |
| Gene Ontology | ribosomal subunit | 21.3793 | 8 | 0.00501159 |
| Gene Ontology | defense response to bacterium | 21.3793 | 8 | 0.00501159 |
| Gene Ontology | serine-type endopeptidase activity | 27.2101 | 12 | 0.00501806 |
| Gene Ontology | neuronal cell body | 32.1574 | 51 | 0.00505004 |
| Gene Ontology | cell body | 32.1574 | 51 | 0.00505004 |
| Gene Ontology | nucleoside binding | 288.003 | 339 | 0.00521729 |
| Gene Ontology | clathrin-coated vesicle | 25.9732 | 43 | 0.00525731 |
| Gene Ontology | induction of programmed cell death | 59.1907 | 84 | 0.00534298 |
| Gene Ontology | adenyl nucleotide binding | 279.875 | 330 | 0.00548673 |
| Gene Ontology | chromatin remodeling complex | 14.8419 | 28 | 0.00551186 |
| Gene Ontology | postsynaptic density | 14.1351 | 27 | 0.00554073 |
| Gene Ontology | ATP binding | 261.499 | 310 | 0.00566214 |
| Gene Ontology | cellular response to organic substance | 47.706 | 70 | 0.00576316 |
| Gene Ontology | regulation of transport | 92.7616 | 123 | 0.00586821 |
| Gene Ontology | serine-type peptidase activity | 31.0972 | 15 | 0.00589341 |
| Gene Ontology | interphase of mitotic cell cycle | 21.556 | 37 | 0.00596931 |
| Gene Ontology | tubulin binding | 17.8456 | 32 | 0.00607488 |
| Gene Ontology | ncRNA metabolic process | 41.6986 | 23 | 0.006076 |
| Gene Ontology | negative regulation of cell communication | 59.5441 | 84 | 0.00633435 |
| Gene Ontology | cell growth | 47.8827 | 70 | 0.00633435 |
| Gene Ontology | skeletal muscle tissue development | 16.4321 | 30 | 0.006378 |
| Gene Ontology | transmembrane receptor protein tyrosine kinase activity | 11.4848 | 23 | 0.00663878 |
| Gene Ontology | epidermal growth factor receptor signaling pathway | 8.12768 | 18 | 0.00670377 |
| Gene Ontology | chromatin modification | 57.2472 | 81 | 0.00711247 |
| Gene Ontology | regulation of myeloid cell apoptosis | 2.29695 | 8 | 0.00714122 |
| Gene Ontology | membrane fraction | 141.174 | 177 | 0.00743061 |
| Gene Ontology | regulation of cell adhesion | 27.2101 | 44 | 0.00749219 |
| Gene Ontology | plasma membrane | 664.173 | 733 | 0.00753812 |
| Gene Ontology | MAP kinase kinase kinase activity | 3.35709 | 10 | 0.00766996 |
| Gene Ontology | ligase activity, forming carbon-nitrogen bonds | 48.236 | 70 | 0.00766996 |
| Gene Ontology | acid-amino acid ligase activity | 43.2887 | 64 | 0.00766996 |
| Gene Ontology | positive regulation of cell differentiation | 45.7624 | 67 | 0.00766996 |
| Gene Ontology | cellular response to endogenous stimulus | 33.5709 | 52 | 0.00766996 |
| Gene Ontology | lateral plasma membrane | 2.82702 | 9 | 0.00768292 |
| Gene Ontology | N-glycan processing | 1.41351 | 6 | 0.00817618 |
| Gene Ontology | catenin complex | 1.41351 | 6 | 0.00817618 |
| Gene Ontology | morphogenesis of embryonic epithelium | 13.7817 | 26 | 0.00817777 |
| Gene Ontology | regulation of myeloid cell differentiation | 13.075 | 25 | 0.00822432 |
| Gene Ontology | morphogenesis of a branching structure | 23.4996 | 39 | 0.00840533 |
| Gene Ontology | peptidyl-threonine modification | 5.12397 | 13 | 0.00847947 |
| Gene Ontology | embryonic development ending in birth or egg hatching | 66.965 | 92 | 0.00850366 |
| Gene Ontology | neural tube closure | 6.3608 | 15 | 0.00854939 |
| Gene Ontology | cellular response to hormone stimulus | 32.1574 | 50 | 0.00855098 |
| Gene Ontology | skeletal muscle organ development | 16.7854 | 30 | 0.00895466 |
| Gene Ontology | regulation of transmembrane receptor protein serine/threonine kinase signaling pathway | 16.7854 | 30 | 0.00895466 |
| Gene Ontology | chordate embryonic development | 66.2583 | 91 | 0.0090568 |
| Gene Ontology | regulation of striated muscle tissue development | 9.01113 | 19 | 0.00914845 |
| Gene Ontology | regulation of Rho protein signal transduction | 19.0824 | 33 | 0.00946413 |
| Gene Ontology | striated muscle cell differentiation | 19.0824 | 33 | 0.00946413 |
| Gene Ontology | exocrine system development | 7.06755 | 16 | 0.00959316 |
| Gene Ontology | vesicle | 126.863 | 160 | 0.00964792 |
| Gene Ontology | insoluble fraction | 146.652 | 182 | 0.00970327 |
| Gene Ontology | regulation of cell shape | 10.4246 | 21 | 0.00970327 |
| Gene Ontology | hexose transport | 10.4246 | 21 | 0.00970327 |
| Gene Ontology | glucose transport | 10.4246 | 21 | 0.00970327 |
| Gene Ontology | organelle part | 899.169 | 973 | 0.00970327 |
| Gene Ontology | mitochondrion | 225.101 | 184 | 0.00996269 |
| Gene Ontology | response to estrogen stimulus | 20.6726 | 35 | 0.0100013 |
| Gene Ontology | intracellular organelle part | 886.801 | 960 | 0.0100547 |
| Gene Ontology | extracellular space | 132.87 | 101 | 0.010072 |
| Gene Ontology | reproductive structure development | 30.0371 | 47 | 0.0100768 |
| Gene Ontology | homophilic cell adhesion | 24.5597 | 40 | 0.0102561 |
| Gene Ontology | positive regulation of locomotion | 24.5597 | 40 | 0.0102561 |
| Gene Ontology | embryonic morphogenesis | 61.4877 | 85 | 0.0103495 |
| Gene Ontology | nucleoplasm | 165.911 | 203 | 0.0103504 |
| Gene Ontology | cell cycle | 177.749 | 216 | 0.0103642 |
| Gene Ontology | transmission of nerve impulse | 74.386 | 100 | 0.0105331 |
| Gene Ontology | endosome | 68.3786 | 93 | 0.0105789 |
| Gene Ontology | regulation of neuron projection development | 19.2591 | 33 | 0.0107886 |
| Gene Ontology | serine C-palmitoyltransferase complex | 1.06013 | 5 | 0.0108087 |
| Gene Ontology | palmitoyltransferase complex | 1.06013 | 5 | 0.0108087 |
| Gene Ontology | skeletal muscle fiber development | 8.48106 | 18 | 0.0108089 |
| Gene Ontology | ribosome biogenesis | 22.9695 | 10 | 0.0108326 |
| Gene Ontology | regulation of growth | 65.9049 | 90 | 0.0109476 |
| Gene Ontology | gamma-catenin binding | 1.94358 | 7 | 0.0110316 |
| Gene Ontology | G1/S transition of mitotic cell cycle | 9.18782 | 19 | 0.0110669 |
| Gene Ontology | caveola | 9.18782 | 19 | 0.0110669 |
| Gene Ontology | aging | 23.1462 | 38 | 0.0110669 |
| Gene Ontology | response to extracellular stimulus | 47.3526 | 68 | 0.0110669 |
| Gene Ontology | regulation of cell projection organization | 23.1462 | 38 | 0.0110669 |
| Gene Ontology | regulation of muscle organ development | 9.18782 | 19 | 0.0110669 |
| Gene Ontology | interphase | 23.1462 | 38 | 0.0110669 |
| Gene Ontology | regulation of cell development | 47.3526 | 68 | 0.0110669 |
| Gene Ontology | tube closure | 6.53748 | 15 | 0.0110669 |
| Gene Ontology | lamellipodium | 14.1351 | 26 | 0.0112091 |
| Gene Ontology | canonical Wnt receptor signaling pathway | 14.1351 | 26 | 0.0112091 |
| Gene Ontology | regulation of cell-cell adhesion | 5.30066 | 13 | 0.0112614 |
| Gene Ontology | dendrite development | 9.89457 | 20 | 0.0113203 |
| Gene Ontology | muscle fiber development | 9.89457 | 20 | 0.0113203 |
| Gene Ontology | ruffle | 13.4283 | 25 | 0.0113454 |
| Gene Ontology | cellular response to stimulus | 194.711 | 234 | 0.0113832 |
| Gene Ontology | Rho guanyl-nucleotide exchange factor activity | 12.7216 | 24 | 0.0113987 |
| Gene Ontology | post-Golgi vesicle-mediated transport | 10.6013 | 21 | 0.0113987 |
| Gene Ontology | monosaccharide transport | 10.6013 | 21 | 0.0113987 |
| Gene Ontology | endosome transport | 12.7216 | 24 | 0.0113987 |
| Gene Ontology | regulation of anti-apoptosis | 7.24424 | 16 | 0.0118697 |
| Gene Ontology | mRNA 3'-UTR binding | 3.00371 | 9 | 0.0119027 |
| Gene Ontology | learning or memory | 19.4358 | 33 | 0.0120394 |
| Gene Ontology | negative regulation of apoptosis | 71.3823 | 96 | 0.0121372 |
| Gene Ontology | regulation of locomotion | 43.4654 | 63 | 0.012391 |
| Gene Ontology | protein serine/threonine phosphatase activity | 7.95099 | 17 | 0.0126978 |
| Gene Ontology | cytoskeleton organization | 93.2917 | 121 | 0.0127239 |
| Gene Ontology | cell proliferation | 206.372 | 246 | 0.013227 |
| Gene Ontology | peptidase inhibitor activity | 28.0935 | 14 | 0.013227 |
| Gene Ontology | respiratory chain | 13.605 | 4 | 0.013227 |
| Gene Ontology | negative regulation of cell death | 73.3258 | 98 | 0.0132365 |
| Gene Ontology | cell-cell signaling | 127.216 | 159 | 0.0132883 |
| Gene Ontology | synaptic transmission | 63.9613 | 87 | 0.0139101 |
| Gene Ontology | microtubule binding | 12.1915 | 23 | 0.0139725 |
| Gene Ontology | response to estradiol stimulus | 11.4848 | 22 | 0.0140356 |
| Gene Ontology | cell junction assembly | 11.4848 | 22 | 0.0140356 |
| Gene Ontology | interspecies interaction between organisms | 60.6042 | 83 | 0.01426 |
| Gene Ontology | hemopoiesis | 56.3637 | 78 | 0.0143568 |
| Gene Ontology | Golgi apparatus part | 95.4119 | 123 | 0.0143568 |
| Gene Ontology | ligase activity | 77.0363 | 102 | 0.0144667 |
| Gene Ontology | rhythmic process | 28.2702 | 44 | 0.0144667 |
| Gene Ontology | endopeptidase inhibitor activity | 26.5033 | 13 | 0.0148282 |
| Gene Ontology | cell-cell junction assembly | 4.24053 | 11 | 0.0148282 |
| Gene Ontology | Ras guanyl-nucleotide exchange factor activity | 15.1952 | 27 | 0.0150924 |
| Gene Ontology | intrinsic to plasma membrane | 213.44 | 253 | 0.0150924 |
| Gene Ontology | extracellular structure organization | 30.7438 | 47 | 0.0150924 |
| Gene Ontology | AP-type membrane coat adaptor complex | 5.47735 | 13 | 0.0151202 |
| Gene Ontology | locomotion | 109.017 | 138 | 0.0158434 |
| Gene Ontology | coated vesicle | 31.6273 | 48 | 0.0158609 |
| Gene Ontology | cytoplasmic vesicle | 121.562 | 152 | 0.0159092 |
| Gene Ontology | response to steroid hormone stimulus | 40.6384 | 59 | 0.0159092 |
| Gene Ontology | mitochondrial matrix | 39.4016 | 23 | 0.0161312 |
| Gene Ontology | ribonucleoprotein complex biogenesis | 34.2776 | 19 | 0.0161312 |
| Gene Ontology | mitochondrial lumen | 39.4016 | 23 | 0.0161312 |
| Gene Ontology | hemopoietic or lymphoid organ development | 60.0742 | 82 | 0.0162119 |
| Gene Ontology | regulation of muscle contraction | 13.075 | 24 | 0.0162485 |
| Gene Ontology | enzyme activator activity | 61.8411 | 84 | 0.0165842 |
| Gene Ontology | induction of apoptosis by extracellular signals | 19.0824 | 32 | 0.0165894 |
| Gene Ontology | insulin receptor signaling pathway | 9.54119 | 19 | 0.0166695 |
| Gene Ontology | vinculin binding | 1.5902 | 6 | 0.0166695 |
| Gene Ontology | secretory granule organization | 1.5902 | 6 | 0.0166695 |
| Gene Ontology | prostate gland growth | 1.5902 | 6 | 0.0166695 |
| Gene Ontology | mitochondrial membrane part | 23.4996 | 11 | 0.0168154 |
| Gene Ontology | protein amino acid dephosphorylation | 24.5597 | 39 | 0.016832 |
| Gene Ontology | negative regulation of programmed cell death | 72.2657 | 96 | 0.016832 |
| Gene Ontology | positive regulation of cellular component movement | 24.5597 | 39 | 0.016832 |
| Gene Ontology | response to nutrient levels | 43.2887 | 62 | 0.0169451 |
| Gene Ontology | negative regulation of signaling pathway | 47.5293 | 67 | 0.0174438 |
| Gene Ontology | activation of MAPKK activity | 4.94729 | 12 | 0.0175307 |
| Gene Ontology | midbody | 4.94729 | 12 | 0.0175307 |
| Gene Ontology | regulation of cell migration | 39.2249 | 57 | 0.0177615 |
| Gene Ontology | protein serine/threonine phosphatase complex | 6.89086 | 15 | 0.0180644 |
| Gene Ontology | primary neural tube formation | 6.89086 | 15 | 0.0180644 |
| Gene Ontology | recycling endosome membrane | 3.1804 | 9 | 0.0180662 |
| Gene Ontology | endonuclease activity | 17.6689 | 7 | 0.0181738 |
| Gene Ontology | cellular respiration | 17.6689 | 7 | 0.0181738 |
| Gene Ontology | dephosphorylation | 31.0972 | 47 | 0.0182799 |
| Gene Ontology | regulation of protein localization | 31.0972 | 47 | 0.0182799 |
| Gene Ontology | response to carbohydrate stimulus | 13.9584 | 25 | 0.018455 |
| Gene Ontology | multivesicular body | 2.65033 | 8 | 0.0190893 |
| Gene Ontology | ion channel inhibitor activity | 2.65033 | 8 | 0.0190893 |
| Gene Ontology | myeloid cell apoptosis | 2.65033 | 8 | 0.0190893 |
| Gene Ontology | neural tube formation | 8.30437 | 17 | 0.0197295 |
| Gene Ontology | regulation of glucose import | 5.65404 | 13 | 0.0198851 |
| Gene Ontology | metal ion transport | 87.6376 | 113 | 0.0199925 |
| Gene Ontology | negative regulation of intracellular protein kinase cascade | 9.71788 | 19 | 0.0203381 |
| Gene Ontology | cytoplasmic mRNA processing body | 4.41722 | 11 | 0.0204146 |
| Gene Ontology | negative regulation of developmental process | 52.1232 | 72 | 0.0205102 |
| Gene Ontology | cell cortex | 23.3229 | 37 | 0.0212469 |
| Gene Ontology | actin cytoskeleton | 49.6495 | 69 | 0.0212469 |
| Gene Ontology | regulation of cellular response to stress | 24.9131 | 39 | 0.0213109 |
| Gene Ontology | endopeptidase regulator activity | 27.0334 | 14 | 0.022079 |
| Gene Ontology | positive regulation of protein amino acid phosphorylation | 22.6162 | 36 | 0.0225969 |
| Gene Ontology | membrane-bounded vesicle | 118.381 | 147 | 0.023035 |
| Gene Ontology | epithelial cell development | 7.06755 | 15 | 0.0232493 |
| Gene Ontology | striated muscle cell development | 12.7216 | 23 | 0.0233321 |
| Gene Ontology | positive regulation of Wnt receptor signaling pathway | 3.88715 | 10 | 0.023626 |
| Gene Ontology | vascular endothelial growth factor receptor signaling pathway | 3.88715 | 10 | 0.023626 |
| Gene Ontology | regulation of skeletal muscle fiber development | 3.88715 | 10 | 0.023626 |
| Gene Ontology | response to mineralocorticoid stimulus | 3.88715 | 10 | 0.023626 |
| Gene Ontology | branching involved in salivary gland morphogenesis | 3.88715 | 10 | 0.023626 |
| Gene Ontology | phospholipid transporter activity | 5.12397 | 12 | 0.0236681 |
| Gene Ontology | histone deacetylase complex | 7.77431 | 16 | 0.02395 |
| Gene Ontology | cell-cell junction organization | 7.77431 | 16 | 0.02395 |
| Gene Ontology | protein transporter activity | 15.0185 | 26 | 0.0246303 |
| Gene Ontology | protein heterodimerization activity | 39.0482 | 56 | 0.0248762 |
| Gene Ontology | cell-matrix adhesion | 21.2027 | 34 | 0.0251507 |
| Gene Ontology | Golgi membrane | 79.5099 | 103 | 0.0253094 |
| Gene Ontology | regulation of molecular function | 191.001 | 226 | 0.0259028 |
| Gene Ontology | kidney development | 18.9057 | 31 | 0.0259772 |
| Gene Ontology | filopodium | 5.83073 | 13 | 0.0261735 |
| Gene Ontology | phosphoprotein binding | 5.83073 | 13 | 0.0261735 |
| Gene Ontology | adult walking behavior | 3.35709 | 9 | 0.0268487 |
| Gene Ontology | estrogen receptor signaling pathway | 3.35709 | 9 | 0.0268487 |
| Gene Ontology | axon regeneration | 3.35709 | 9 | 0.0268487 |
| Gene Ontology | sequence-specific DNA binding | 114.494 | 142 | 0.0272132 |
| Gene Ontology | negative regulation of cell differentiation | 42.582 | 60 | 0.0272132 |
| Gene Ontology | basement membrane | 12.8983 | 23 | 0.0272509 |
| Gene Ontology | central nervous system neuron differentiation | 12.8983 | 23 | 0.0272509 |
| Gene Ontology | protein amino acid autophosphorylation | 15.902 | 27 | 0.0272509 |
| Gene Ontology | leukocyte differentiation | 34.2776 | 50 | 0.0275462 |
| Gene Ontology | regulation of cellular component movement | 43.4654 | 61 | 0.027569 |
| Gene Ontology | positive regulation of cell migration | 22.9695 | 36 | 0.0284926 |
| Gene Ontology | regulation of cell growth | 38.5182 | 55 | 0.0289142 |
| Gene Ontology | lipid transport | 28.6236 | 43 | 0.0289211 |
| Gene Ontology | mitochondrial part | 109.37 | 84 | 0.0289489 |
| Gene Ontology | response to organic cyclic substance | 27.0334 | 41 | 0.0294751 |
| Gene Ontology | channel inhibitor activity | 2.82702 | 8 | 0.0296281 |
| Gene Ontology | regulation of vascular endothelial growth factor receptor signaling pathway | 2.82702 | 8 | 0.0296281 |
| Gene Ontology | cell junction organization | 14.4885 | 25 | 0.0296281 |
| Gene Ontology | mitochondrial respiratory chain | 12.3682 | 4 | 0.0297705 |
| Gene Ontology | transmembrane receptor protein serine/threonine kinase signaling pathway | 29.507 | 44 | 0.0299666 |
| Gene Ontology | mRNA cap binding complex | 1.76689 | 6 | 0.0307302 |
| Gene Ontology | axon cargo transport | 2.29695 | 7 | 0.0307302 |
| Gene Ontology | asymmetric protein localization | 1.76689 | 6 | 0.0307302 |
| Gene Ontology | intercalated disc | 2.29695 | 7 | 0.0307302 |
| Gene Ontology | cytoplasmic membrane-bounded vesicle | 115.024 | 142 | 0.0307302 |
| Gene Ontology | guanyl nucleotide binding | 67.8485 | 89 | 0.0307302 |
| Gene Ontology | clathrin adaptor complex | 5.30066 | 12 | 0.0307302 |
| Gene Ontology | regulation of BMP signaling pathway | 5.30066 | 12 | 0.0307302 |
| Gene Ontology | G1/S DNA damage checkpoint | 2.29695 | 7 | 0.0307302 |
| Gene Ontology | regulation of heat generation | 1.76689 | 6 | 0.0307302 |
| Gene Ontology | positive regulation of heat generation | 1.76689 | 6 | 0.0307302 |
| Gene Ontology | guanyl ribonucleotide binding | 67.8485 | 89 | 0.0307302 |
| Gene Ontology | regulation of activin receptor signaling pathway | 2.29695 | 7 | 0.0307302 |
| Gene Ontology | RNA cap binding complex | 1.76689 | 6 | 0.0307302 |
| Gene Ontology | RNA polyadenylation | 2.29695 | 7 | 0.0307302 |
| Gene Ontology | cell-cell contact zone | 2.29695 | 7 | 0.0307302 |
| Gene Ontology | nucleoplasm part | 106.013 | 132 | 0.0307302 |
| Gene Ontology | positive regulation of gene expression, epigenetic | 1.76689 | 6 | 0.0307302 |
| Gene Ontology | elevation of cytosolic calcium ion concentration involved in G-protein signaling coupled to IP3 second messenger | 1.76689 | 6 | 0.0307302 |
| Gene Ontology | regulation of intracellular protein kinase cascade | 59.1907 | 79 | 0.0310186 |
| Gene Ontology | cell projection part | 63.7846 | 84 | 0.0346886 |
| Gene Ontology | smooth muscle contraction | 10.9547 | 20 | 0.0347632 |
| Gene Ontology | regulation of smooth muscle contraction | 6.71417 | 14 | 0.034904 |
| Gene Ontology | regulation of muscle cell differentiation | 6.71417 | 14 | 0.034904 |
| Gene Ontology | developmental growth | 28.977 | 43 | 0.0349311 |
| Gene Ontology | regulation of binding | 37.2813 | 53 | 0.0353271 |
| Gene Ontology | clathrin coated vesicle membrane | 13.9584 | 24 | 0.0353992 |
| Gene Ontology | regulation of cell cycle | 79.6866 | 102 | 0.0353992 |
| Gene Ontology | muscle cell development | 13.9584 | 24 | 0.0353992 |
| Gene Ontology | specific RNA polymerase II transcription factor activity | 7.42093 | 15 | 0.0356969 |
| Gene Ontology | antral ovarian follicle growth | 0.883444 | 4 | 0.0360081 |
| Gene Ontology | polynucleotide adenylyltransferase activity | 0.883444 | 4 | 0.0360081 |
| Gene Ontology | serine C-palmitoyltransferase activity | 0.883444 | 4 | 0.0360081 |
| Gene Ontology | transmembrane-ephrin receptor activity | 0.883444 | 4 | 0.0360081 |
| Gene Ontology | GTP binding | 65.7282 | 86 | 0.0360081 |
| Gene Ontology | ethanolamine metabolic process | 0.883444 | 4 | 0.0360081 |
| Gene Ontology | phosphatidylethanolamine biosynthetic process | 0.883444 | 4 | 0.0360081 |
| Gene Ontology | humoral immune response | 15.0185 | 6 | 0.0360081 |
| Gene Ontology | C-palmitoyltransferase activity | 0.883444 | 4 | 0.0360081 |
| Gene Ontology | oxidoreductase activity, acting on NADH or NADPH, quinone or similar compound as acceptor | 8.83444 | 2 | 0.0360081 |
| Gene Ontology | cytokinetic process | 0.883444 | 4 | 0.0360081 |
| Gene Ontology | RPTP-like protein binding | 0.883444 | 4 | 0.0360081 |
| Gene Ontology | extracellular organelle | 0.883444 | 4 | 0.0360081 |
| Gene Ontology | protein kinase B binding | 0.883444 | 4 | 0.0360081 |
| Gene Ontology | regulation of transcription from RNA polymerase II promoter in response to stress | 0.883444 | 4 | 0.0360081 |
| Gene Ontology | ethanolamine biosynthetic process | 0.883444 | 4 | 0.0360081 |
| Gene Ontology | phosphatidylethanolamine metabolic process | 0.883444 | 4 | 0.0360081 |
| Gene Ontology | dichotomous subdivision of terminal units involved in salivary gland branching | 0.883444 | 4 | 0.0360081 |
| Gene Ontology | extracellular membrane-bounded organelle | 0.883444 | 4 | 0.0360081 |
| Gene Ontology | intracellular organelle lumen | 325.991 | 368 | 0.0360081 |
| Gene Ontology | ciliary neurotrophic factor-mediated signaling pathway | 0.883444 | 4 | 0.0360081 |
| Gene Ontology | serine-type endopeptidase inhibitor activity | 16.4321 | 7 | 0.0361346 |
| Gene Ontology | renal system development | 19.4358 | 31 | 0.0362138 |
| Gene Ontology | integral to plasma membrane | 209.553 | 244 | 0.0362677 |
| Gene Ontology | RNA stabilization | 3.53378 | 9 | 0.0363991 |
| Gene Ontology | regulation of erythrocyte differentiation | 3.53378 | 9 | 0.0363991 |
| Gene Ontology | mRNA stabilization | 3.53378 | 9 | 0.0363991 |
| Gene Ontology | mRNA binding | 12.5449 | 22 | 0.0370912 |
| Gene Ontology | structural molecule activity | 107.073 | 83 | 0.0371546 |
| Gene Ontology | immune system development | 64.138 | 84 | 0.0379101 |
| Gene Ontology | system process | 288.356 | 250 | 0.0379101 |
| Gene Ontology | regulation of muscle system process | 14.8419 | 25 | 0.0379101 |
| Gene Ontology | sodium ion transport | 23.4996 | 36 | 0.0380228 |
| Gene Ontology | lipid localization | 32.5107 | 47 | 0.0382483 |
| Gene Ontology | regulation of kinase activity | 69.4387 | 90 | 0.0383557 |
| Gene Ontology | vacuolar transport | 5.47735 | 12 | 0.0387207 |
| Gene Ontology | organelle lumen | 332.352 | 374 | 0.0391419 |
| Gene Ontology | oxidoreductase activity, acting on NADH or NADPH | 14.8419 | 6 | 0.0393582 |
| Gene Ontology | organellar ribosome | 8.65775 | 2 | 0.0396924 |
| Gene Ontology | mitochondrial ribosome | 8.65775 | 2 | 0.0396924 |
| Gene Ontology | mitotic cell cycle | 86.4008 | 109 | 0.0398863 |
| Gene Ontology | cellular protein catabolic process | 61.6644 | 81 | 0.0398863 |
| Gene Ontology | epithelial cell proliferation | 18.0223 | 29 | 0.0401234 |
| Gene Ontology | cell aging | 6.18411 | 13 | 0.04066 |
| Gene Ontology | regulation of glucose transport | 6.18411 | 13 | 0.04066 |
| Gene Ontology | regulation of system process | 55.657 | 74 | 0.0411461 |
| Gene Ontology | protein monoubiquitination | 3.00371 | 8 | 0.0415026 |
| Gene Ontology | vesicle fusion | 3.00371 | 8 | 0.0415026 |
| Gene Ontology | clathrin coat | 6.89086 | 14 | 0.0418218 |
| Gene Ontology | protein stabilization | 6.89086 | 14 | 0.0418218 |
| Gene Ontology | response to nutrient | 30.2138 | 44 | 0.0419728 |
| Gene Ontology | cell-substrate junction | 17.3155 | 28 | 0.0422684 |
| Gene Ontology | cytosolic ribosome | 13.2517 | 5 | 0.0422817 |
| Gene Ontology | SMAD binding | 8.30437 | 16 | 0.0422817 |
| Gene Ontology | kinase binding | 36.928 | 52 | 0.0426773 |
| Gene Ontology | membrane-enclosed lumen | 338.712 | 380 | 0.0430779 |
| Gene Ontology | secondary active transmembrane transporter activity | 34.4543 | 49 | 0.0436794 |
| Gene Ontology | common-partner SMAD protein phosphorylation | 0.530066 | 3 | 0.04411 |
| Gene Ontology | retrograde axon cargo transport | 0.530066 | 3 | 0.04411 |
| Gene Ontology | cardiac muscle adaptation | 0.530066 | 3 | 0.04411 |
| Gene Ontology | insulin-like growth factor binding protein complex | 0.530066 | 3 | 0.04411 |
| Gene Ontology | protein amino acid O-linked glycosylation via serine | 0.530066 | 3 | 0.04411 |
| Gene Ontology | protein amino acid O-linked glycosylation via threonine | 0.530066 | 3 | 0.04411 |
| Gene Ontology | negative regulation of vascular endothelial growth factor receptor signaling pathway | 0.530066 | 3 | 0.04411 |
| Gene Ontology | positive regulation of activin receptor signaling pathway | 0.530066 | 3 | 0.04411 |
| Gene Ontology | response to vitamin E | 0.530066 | 3 | 0.04411 |
| Gene Ontology | positive regulation of axon regeneration | 0.530066 | 3 | 0.04411 |
| Gene Ontology | thyroid-stimulating hormone-secreting cell differentiation | 0.530066 | 3 | 0.04411 |
| Gene Ontology | positive regulation of neuron projection regeneration | 0.530066 | 3 | 0.04411 |
| Gene Ontology | peptidyl-amino acid modification | 43.8188 | 60 | 0.044191 |
| Gene Ontology | proteolysis involved in cellular protein catabolic process | 61.1343 | 80 | 0.0443139 |
| Gene Ontology | myeloid leukocyte differentiation | 14.3118 | 24 | 0.0447354 |
| Gene Ontology | positive regulation of cell-cell adhesion | 2.47364 | 7 | 0.045279 |
| Gene Ontology | filamentous actin | 2.47364 | 7 | 0.045279 |
| Gene Ontology | MAP kinase phosphatase activity | 2.47364 | 7 | 0.045279 |
| Gene Ontology | regulation of cell proliferation | 150.539 | 179 | 0.045279 |
| Gene Ontology | ATP synthesis coupled electron transport | 10.0713 | 3 | 0.045279 |
| Gene Ontology | mitochondrial ATP synthesis coupled electron transport | 10.0713 | 3 | 0.045279 |
| Gene Ontology | transcription repressor binding | 2.47364 | 7 | 0.045279 |
| Gene Ontology | regulation of skeletal muscle tissue development | 4.94729 | 11 | 0.0454524 |
| Gene Ontology | vascular endothelial growth factor receptor activity | 1.41351 | 5 | 0.046041 |
| Gene Ontology | regulation of protein stability | 10.6013 | 19 | 0.046041 |
| Gene Ontology | gas homeostasis | 1.41351 | 5 | 0.046041 |
| Gene Ontology | negative regulation of Rho protein signal transduction | 1.41351 | 5 | 0.046041 |
| Gene Ontology | identical protein binding | 123.152 | 149 | 0.046041 |
| Gene Ontology | neurotrophin binding | 1.41351 | 5 | 0.046041 |
| Gene Ontology | regulation of lipid kinase activity | 1.41351 | 5 | 0.046041 |
| Gene Ontology | apical protein localization | 1.41351 | 5 | 0.046041 |
| Gene Ontology | ephrin receptor binding | 1.41351 | 5 | 0.046041 |
| Gene Ontology | embryonic foregut morphogenesis | 1.41351 | 5 | 0.046041 |
| Gene Ontology | neurofilament cytoskeleton organization | 1.41351 | 5 | 0.046041 |
| Gene Ontology | neurofilament cytoskeleton | 1.41351 | 5 | 0.046041 |
| Gene Ontology | regulation of vesicle-mediated transport | 22.2628 | 34 | 0.046041 |
| Gene Ontology | MLL5-L complex | 1.41351 | 5 | 0.046041 |
| Gene Ontology | establishment of protein localization in plasma membrane | 1.41351 | 5 | 0.046041 |
| Gene Ontology | establishment of protein localization in membrane | 1.41351 | 5 | 0.046041 |
| Gene Ontology | cell projection assembly | 17.4922 | 28 | 0.0460508 |
| Gene Ontology | secretion by cell | 69.262 | 89 | 0.0465441 |
| Gene Ontology | ncRNA processing | 34.1009 | 21 | 0.0465441 |
| Gene Ontology | in utero embryonic development | 39.755 | 55 | 0.0468472 |
| Gene Ontology | phosphoprotein phosphatase activity | 29.6837 | 43 | 0.0468472 |
| Gene Ontology | non-G-protein coupled 7TM receptor activity | 1.94358 | 6 | 0.0468472 |
| Gene Ontology | salivary gland development | 5.65404 | 12 | 0.0468472 |
| Gene Ontology | phosphatase activity | 44.8789 | 61 | 0.0468472 |
| Gene Ontology | regulation of Cdc42 protein signal transduction | 1.94358 | 6 | 0.0468472 |
| Gene Ontology | methyltransferase complex | 9.89457 | 18 | 0.0468472 |
| Gene Ontology | histone methyltransferase complex | 9.89457 | 18 | 0.0468472 |
| Gene Ontology | Wnt receptor activity | 1.94358 | 6 | 0.0468472 |
| Gene Ontology | regulation of Cdc42 GTPase activity | 1.94358 | 6 | 0.0468472 |
| Gene Ontology | early endosome to late endosome transport | 1.94358 | 6 | 0.0468472 |
| Gene Ontology | inactivation of MAPK activity | 3.71046 | 9 | 0.0474405 |
| Gene Ontology | neuron projection regeneration | 3.71046 | 9 | 0.0474405 |
| Gene Ontology | nucleosome | 11.4848 | 4 | 0.0477486 |
| Gene Ontology | RNA localization | 18.3756 | 29 | 0.0486256 |
| Gene Ontology | regulation of transferase activity | 72.089 | 92 | 0.0486619 |
| Gene Ontology | cytosolic calcium ion transport | 8.48106 | 16 | 0.0486619 |
| Gene Ontology | skeletal system development | 67.6718 | 87 | 0.0487316 |
| Gene Ontology | chromatin binding | 31.4506 | 45 | 0.0490601 |
| Gene Ontology | development of primary female sexual characteristics | 14.4885 | 24 | 0.0494357 |
| Gene Ontology | cellular macromolecule catabolic process | 84.6339 | 106 | 0.0496675 |
